# A comprehensive review of methods for valorisation and production of bioproducts from various cassava wastes

**DOI:** 10.1039/d6ra04237f

**Published:** 2026-08-24

**Authors:** Tammires L. C. Santana, Jude A. Onwudili, Carine T. Alves, Taíse B. de Jesus

**Affiliations:** a Program in Modeling in Earth and Environmental Sciences (PPGM), Department of Exact Sciences (DEXA), State University of Feira de Santana Feira de Santana Bahia Brazil; b Energy and Bioproducts Research Institute (EBRI), College of Engineering and Physical Sciences, Aston University Aston Triangle Birmingham UK; c Federal University of Recôncavo of Bahia, Center for Science and Technology in Energy and Sustainability (CETENS) Feira de Santana Bahia Brazil 14121203@discente.uefs.br

## Abstract

Cassava is a key tropical crop; however, its processing generates large volumes of residues, including peels, bagasse, leaves, stems, and wastewater. Although these wastes are rich in starch, fibre, and lignocellulosic components with high biotechnological potential, they remain largely underutilised. In this context, this review examines current strategies for the recovery and valorisation of cassava processing residues, encompassing physical, chemical, biological, and enzymatic pretreatments, as well as emerging technologies aimed at biofuel and bioproduct generation. Hydrothermal pretreatment stands out for offering a balanced performance between efficiency and inhibitor formation, while acid pretreatment maximises hemicellulose solubilisation but generates higher levels of toxic compounds. Alkaline pretreatments promote effective delignification and enhance enzymatic accessibility, albeit producing effluents that require further treatment. Biological and enzymatic approaches present environmental advantages and low inhibitor formation, although their application is limited by slower kinetics. Emerging technologies, such as ultrasound and non-thermal plasma, show promising potential to enhance sugar release and reduce hydrolysate toxicity, despite the limited number of studies specifically focused on cassava residues. Overall, the integration of complementary routes emerges as a robust strategy to maximise the production of bioethanol, biogas, biohydrogen, and organic acids from cassava wastes. Biorefinery models consistently demonstrate significant energy gains and reduced environmental impacts, reinforcing cassava residues as a strategic platform for circular economy applications. Consequently, the integrated valorisation of cassava processing wastes represents a relevant opportunity to enhance technological sustainability and environmental performance across cassava value chains.

## Abstract 2

Cassava residues are abundant yet underutilised biomass resources. This review examines pretreatment methods, valorisation pathways and biorefinery strategies for producing biofuels, biochemicals and value-added products.

## Graphical abstract

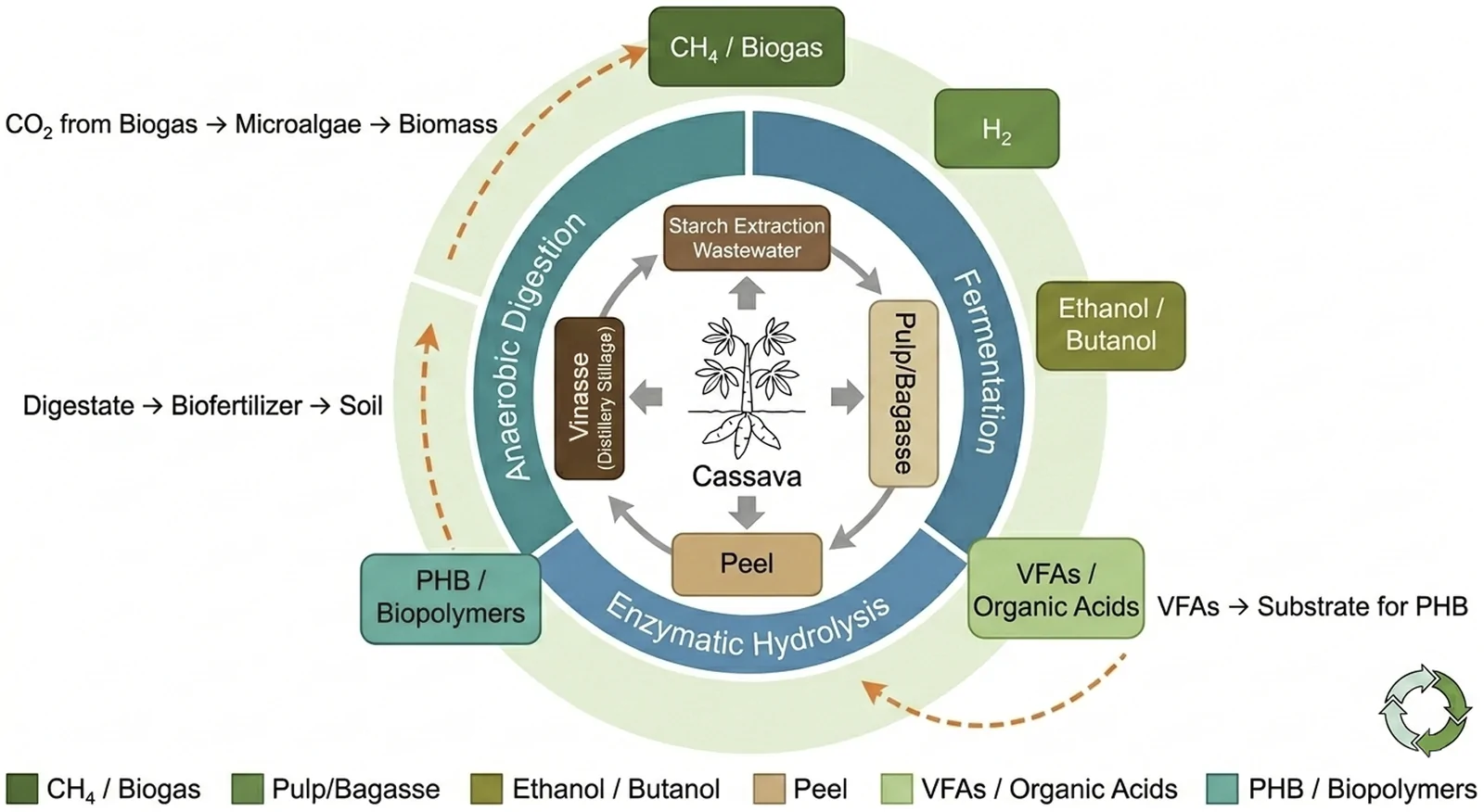

## 1. Introduction

Cassava (*Manihot esculenta crantz*) plays a central role in food security and socioeconomic development in tropical regions. It is a major source of carbohydrates and a strategic input for various agro-industrial segments. As one of the largest sources of carbohydrates in the tropics, along with rice and corn, the crop sustains the food security of millions of people in developing countries.^1,2^ Its tuberous roots contain between 30 and 40% dry matter, of which 25 to 30% is starch. In addition, it is a relevant source of calcium, iron, vitamin C, and folate.^1,2^ The leaves, in turn, have a high protein content and are rich in vitamins A and C, representing underutilised fractions with significant nutritional potential.^3,4^

Its resilience to drought conditions, marginal soils, and high temperatures, tolerating pH ranges between 4.0 and 8.0, ensure that it maintains productivity where other crops fail. It can remain stored in the soil for up to 24 months as a food reserve in crises, which gives it a strategic role in climate adaptation scenarios, justifying its designation as a “crop of drought, war, and famine”.^4,5^ World cassava production exceeded 315 million tonnes in 2021, with an African contribution of approximately 65%. Nigeria is the world's largest producer, providing 20% of global production and with an average annual growth exceeding 4%.^1,6^ In addition to its relevance in human and animal nutrition, the crop has been used as an essential industrial raw material. It is to produce starches, flour, and industrial derivatives, responding to growing global demands in sectors such as food, bioenergy, and biotechnology.^7,8^

Cassava processing generates large volumes of solid and liquid residues, accounting for up to 55% of the total processed biomass.^9^ In Nigerian rural garri production, peels, starchy effluents, and fibrous residues represent approximately 52.6% of processed tuber mass.^6^ Cassava stalks discarded after harvest constitute one of Nigeria's largest agricultural waste streams, exceeding residues from other major crops. These residues contribute to a national bioenergy potential of 1.09 × 10^12^ MJ, with cassava as a major contributor.^10^ Their composition favours bioenergy conversion, containing about 21.43% cellulose, 11.62% hemicellulose, and 42% starch on a dry basis.^3,6^

When discarded untreated, these residues cause serious environmental impacts, including soil acidification, contamination of water bodies, the release of toxic compounds, and greenhouse gas emissions.^11^ Most cassava species contain cyanogenic compounds, especially free cyanide (CN^−^) and hydrocyanic acid (HCN), which are released during processing. These compounds represent a documented ecotoxicological risk in multiple tropical countries, with free CN concentrations in cassava wastewater reaching averages of 267 ± 80 mg L^−1^ and levels in peels up to 370 mg kg^−1^.^12,13^

Exploration of microbial biodegradation routes for cyanide, including aerobic and anaerobic processes using native microbial consortia is being exploited. It emerges as a sustainable alternative to conventional physicochemical treatment, with removal efficiencies exceeding 90% when optimised. Technologies such as electro-biodegradation, microbial fuel cells (MFCs), and immobilized enzymes can remove up to 99.7% of cyanide. MFCs can also simultaneously generating electricity or value-added products.^12^ Low-cost bioremediation strategies have demonstrated reductions exceeding 55% in cyanide concentration in effluents and potential for revitalizing contaminated soils.^14^ These include the application of nutrient-rich biomass with an alkaline character possessing nitrogen (9.54%) and phosphorus (8.50%).

Cassava processing effluents can significantly alter soil pH, conductivity, and microbiota, accumulating heavy metals whose anthropogenic fraction can exceed 90% in soils adjacent to artisanal mills. This pattern has been identified for cadmium, zinc, and manganese in producing communities in Delta State, Nigeria.^15–17^ Socioeconomic surveys in Ogun State, another area of Nigeria reveal that 90.5% of processors dispose of waste in open landfills, while only 1.6% use it as fertilizer.^7^ However, 74.7% of these cassava processors demonstrate a willingness to adopt improved management systems.^7^

Despite environmental challenges, a growing body of research shows that cassava harvesting and processing residues have high potential for technological valorisation. Cassava waste is rich in starch, fiber, fermentable sugars, proteins, and minerals. Thus, allowing their conversion into a wide range of high-value-added bioproducts, such as biogas, bioethanol, butanol, biohydrogen, organic acids, bioplastics, adsorbents, biosurfactants, enzymes, and fertilizers.^11,18^

The potential of acidogenic fermentations for producing volatile fatty acids (VFAs) as precursors of polyhydroxyalkanoates (PHAs) and other high-value biopolymers is also highlighted. This route integrates waste valorisation and the replacement of conventional plastics.^19^ Advances in hybrid thermochemical–biological processes, pyrolysis, gasification, and microwave-assisted hydrolysis have further increased conversion efficiency and reduced the environmental impact of processing.^9,20,21^

Therefore, cassava-based biorefinery models are widely recognized as effective strategies for advancing circular economy transitions. Sánchez *et al.*^22^ showed that anaerobic digestion and cogeneration can render starch industries energy-positive, achieving efficiencies above 85% and producing up to 19 N m^3^ CH_4_ per t cassava. This configuration also generates liquid biofertilizers supplying approximately 350 kg N, 50 kg P, and 400 kg K per ha per year. Integrated systems valorising peels, effluents, and stem biochar combined with microbial fuel cells reached 94.33 mW m^−2^ and increased cumulative biogas by 102%.^9^ Scenario analyses for cassava starch industries indicate high profitability, with integrated feed and protein routes generating up to USD 8.85 million per cycle and avoiding 57.45 t CO_2_eq.^23^ Comparative assessments show cassava peel exhibits high renewability (94%) and lower climate impact than corn straw, requiring less intense pretreatment.^24^ Life-cycle studies confirm that integrated valorisation of cassava waste outperforms conventional disposal in both environmental and climate metrics.^19,25^

In this context, cassava has transitioned from a subsistence staple to a important axis for sustainable biotechnological value chains. This review evaluates recovery and valorisation routes for waste generated along the cassava processing chain. It addresses physical, chemical, biological, and integrated processes for producing value-added bioproducts. The study identifies advantages, limitations, scientific gaps, and innovation opportunities, contributing to clean technologies and the industrial consolidation of cassava-based biorefineries.

## 2. Overview of cassava origin and internationalisation

Cassava originating in Latin America, is one of the oldest cultivated plants on the continent, playing a central role in the subsistence, culture, and environmental transformation of various societies.^26^ Archaeological evidence indicates that its cultivation began more than 10 000 years ago in southwestern Amazonia. There, it contributed to the formation of anthropogenic forest islands, areas of modified vegetation that functioned as nuclei of fertility and diversity.^27^

The species exhibits wide genetic diversity, reflected in the popular classification between “sweet” and “bitter” varieties. Sweet varieties have low cyanogen contents and are generally consumed after simple preparation, such as cooking. Bitter varieties, in contrast, have a high content of cyanogenic compounds, requiring more complex processing to ensure food safety.^28^ However, this popular distinction does not always correspond to the actual levels of toxicity, as environmental factors, such as altitude, directly influence this expression.^29^

Studies conducted in southeastern Nigeria evaluated twenty-eight cassava genotypes, classified according to their cyanogenic compound content. Sweet varieties showed between 15–50 mg HCN per kg of fresh weight, while bitter varieties ranged from 15 to 400 mg HCN per kg. Although they did not differ significantly in productivity, the bitter varieties demonstrated greater tolerance to cassava mosaic disease. The presence of cyanogenic glycosides may therefore contribute to pest resistance, but does not guarantee agronomic superiority.^30^

Cassava domestication, like that of other crops, occurred through parallel and convergent processes across regions, revealing shared evolutionary patterns alongside cultural particularities.^31^ Initially originated in Latin America, cassava spread globally *via* Portuguese colonial trade, becoming a staple food in tropical regions, enslaved populations, and trading posts.^32,33^

African countries currently dominate global cassava production, while Latin American nations hold secondary positions.^34^ After its introduction, cassava became a pillar of African food security, especially in socially and economically vulnerable contexts.^32^ In West Africa's Igbo society, cassava was incorporated as an essential crop alongside yam, traditionally regarded as the dominant staple. Cassava came to be known as the “mother of all crops” due to its capacity to sustain communities during periods of crisis and scarcity.^35^

In the global scenario, marked by the increasing homogenisation of food systems, cassava stands out as a strategic alternative. Due to its hardiness and adaptability, cassava contributes to the quality of production systems and food security.^36^ In Latin America, the species also plays a relevant role in sustainable agricultural practices, especially using biological control agents. Integrated management programs have reduced the use of chemical pesticides, preserved natural enemies, and mitigated environmental impacts. As a basic food and socioeconomic crop, cassava demands low-cost and highly effective solutions, becoming a symbol of the integration between traditional knowledge and applied science.^37^

## 3. Cassava production and cultivated area

**Fig. 1:**
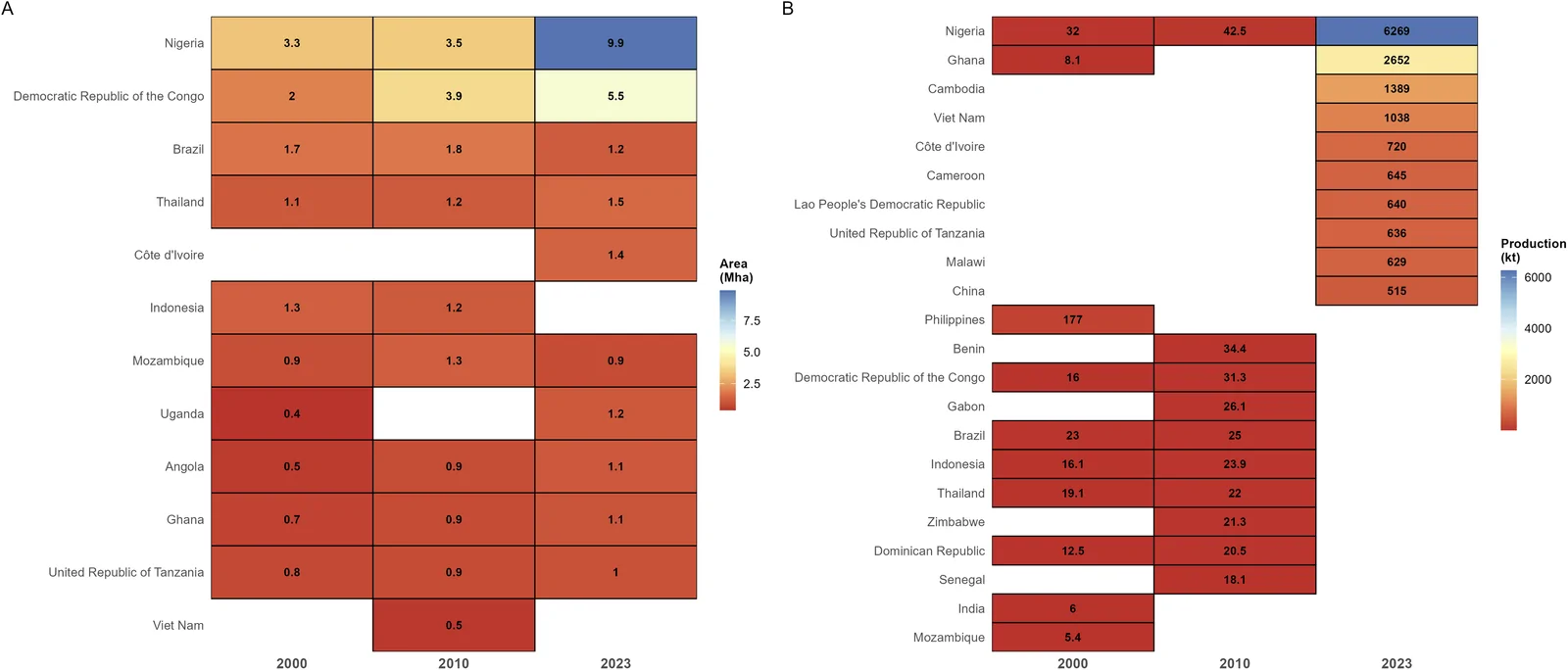
Area harvested (A) and production (B) of cassava in the top 10 producing countries for the years 2000, 2010, and 2023. Source: Authors' elaboration based on FAOSTAT data (FAO, 2023).

Data obtained from FAO^38^ database show major changes in global cassava production between 2000, 2010, and 2023, accompanied by shifts in cultivated area (Fig. 1a and b). Nigeria consolidated leadership through area expansion, increasing cultivation from 3.3 to 9.9 million hectares, while production rose sharply over the period. A similar land-expansion trend occurred in the Democratic Republic of Congo, with cultivated area increasing from 2 to 5.5 million hectares. Conversely, Brazil reduced cultivated area but recovered production through planting density, mechanisation, and improved varieties, highlighting gains *via* technological intensification.^39^

Africa accounts for over 60% of global cassava production, mainly through cultivated area expansion, an unsustainable strategy without concomitant productivity gains. In the Democratic Republic of Congo, yield increases result from integrated soil fertility management, improved germplasm, optimized planting arrangements, and moderate fertiliser use.^40^ Asia produces nearly 30% of global cassava using only 13% of cultivated area, reflecting industrial utilization and advanced agronomic practices. These contrasts show that territorial expansion alone does not ensure competitiveness or sustainability, highlighting the need for productivity-enhancing technologies with lower environmental impacts.^41^

Despite Africa's production leadership, the continent contributes less than 5% of global starch output, reflecting post-harvest losses and limited industrialisation of cassava chains.^41^ This constraint is exacerbated by post-harvest physiological deterioration, which begins shortly after harvest and involves oxidative reactions and phenolic accumulation. Additionally, mite infestations can reduce cassava yields by up to 80%, further undermining productivity and value-chain integration.^42,43^

Investments in research and technological development have markedly improved agricultural productivity worldwide. Technologies promoted by CGIAR were adopted on over 10 million hectares between 2016 and 2020, notably in Sub-Saharan Africa, generating significant economic gains.^44^ India provides a strong example, where technologies disseminated between 1966 and 2000 generated an economic surplus exceeding USD 37.23 million and an internal rate of return of 104%.^45^ In Brazil, Embrapa and universities have led genetic improvement and sustainable management research, developing pest-resistant varieties adapted to diverse environments. Bibliometric analysis confirms Brazil's leadership in cassava genetics, with Embrapa as the most productive institution, focusing on germplasm conservation and molecular breeding.^46^ However, productivity gains alone do not ensure farmer income without value addition along the production chain. Evidence from Nigeria shows that farmers investing in processing or marketing achieved higher technical efficiency, supported by access to extension services and credit.^47^ Brazilian cassava industrialisation, including starch, flour, and bioethanol production, demonstrates that value addition is essential for competitiveness and smallholder income generation. Additionally, economic analyses indicate that replacing tobacco with cassava in Uganda could significantly increase net income and reduce rural poverty, reinforcing the crop's social importance.^48^

Full utilisation of cassava production, including residues such as peels, enhances energy recovery through bioethanol and biomethane generation. Optimisation models in southwestern Nigeria identify Lagos as a strategic biorefinery hub, supporting cleaner energy transitions.^49^ Cassava's tolerance to adverse climates, demonstrated in semi-arid Zimbabwe and Mozambique, confirms its relevance under climate change scenarios.^50,51^IPCC projections indicate warming and reduced rainfall, yet cassava shows higher resilience and potential range expansion compared to other staple crops.^52,53^ However, territorial expansion alone increases environmental pressure and vulnerability to pests, diseases, and cyanogenic toxicity.^54,55^ Sustainable intensification strategies, including irrigation, genetic improvement, pest management, and value-chain policies, are therefore essential.^5,56^ Recent genetic advances, such as waxy-starch cassava varieties, further expand industrial applications without significantly compromising yield.^57^

### 3.1. Types of waste generated and their nutritional compositions

The cassava production chain generates substantial residues throughout harvesting and processing, creating environmental challenges and opportunities for agro-industrial valorisation.^58,59^ Physicochemical characterisation shows strong compositional heterogeneity, influenced by plant fraction, processing method, and post-harvest handling conditions.^60–62^

Cassava residues include vegetative parts, root system residues, industrial by-products, and discarded tubers, generated across agricultural and processing stages.^58,63^ These fractions differ in protein, ash, fibre, residual starch, and cyanogenic compounds, determining their suitability for specific technological and biotechnological applications.^64,65^

**Fig. 2:**
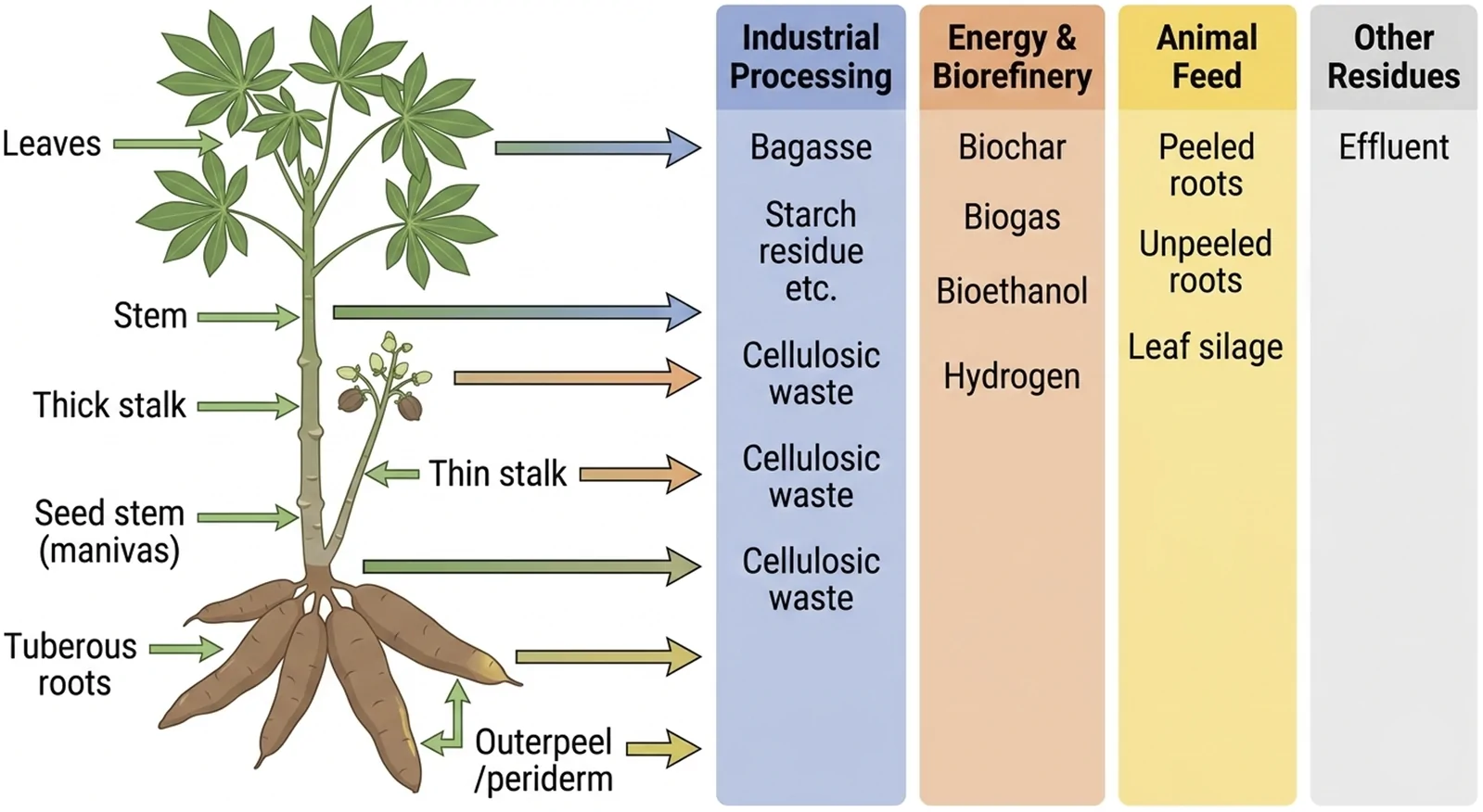
Schematic overview of the main residues and by-products generated along the cassava production and processing chain and their primary valorisation pathways, complementing the compositional data presented in Table 1. Prepared by the author based on Idris *et al.*,^61^ Veiga *et al.*,^58^ Fanelli *et al.*,^62^ Schwantes *et al.*,^85^ Wijitkosum and Jiwnok,^86^ and Aro *et al.*^63^

The compositional heterogeneity of cassava residues depends on plant fraction and processing type.^59,60,62^Table 1 compiles bromatological data from literature for different cassava fractions and by-products, showing variations in crude protein, ash, fiber, and cyanogenic compound contents. Fig. 2 schematically illustrates the origin of each residual fraction within the plant and its main valorisation pathways.

**Table 1:** Bromatological composition of residues from the cassava production chain^a^

Waste	Matter dry (%)	Crude protein (%)	Ash (%)	Fibers (%)	Cyanogenic compounds (g L^−1^)	Authors
Bagasse	N/A	N/A	6.2	N/A	N/A	66
Bagasse	87.0	N/A	N/A	Cellulose: 13.5	N/A	67
				Hemicellulose: 5.8		
				Lignin: 2.8		
Bagasse	N/A	N/A	N/A	Cellulose: 19.3	N/A	68
				Lignin: 4.1		
				Starch: 61.6		
Bagasse	N/A	N/A	N/A	Cellulose: 6.7	N/A	69
				Lignin: 2.0		
				Polysaccharides(Starch + hemicellulose): 89.9		
				Starch: 45.0		
Bagasse	34.8	0.6	0.2	Fiber 4.3	N/A	59
				Starch: 64.3		
				Sugars: 7.8		
Cassava cellulosic waste	N/A	0.8	2.0	Fibre: 15.8	N/A	70
				Starch: 60.8		
Cassava waste	81.3	N/A	N/A	Cellulose: 26.3	N/A	71
				Hemicellulose: 17.6		
				Lignin: 7.0		
Cassava waste	95.6	8.8	N/A	Cellulose: 24.9	N/A	72
				Hemicellulose: 17.8		
				Lignin: 12.3		
Cassava wastewater	N/A	8.4	N/A	Cellulose: 24.9	N/A	72
				Hemicellulose: 17.8		
				Lignin: 12.3		
Leaf	91.4	20.2	7.4	21.8	112.2	73
Leaf	6.1	28.0	7.3	21.4	2 × 10^−2^	61
Leaf	88.0	20.0	2.5	Cellulose 17.3	N/A	74
				Hemicellulose 27.7		
				Lignin: 20.1		
				Starch: 2.4		
Peel	N/A	N/A	N/A	Cellulose: 4.8	N/A	69
				Lignin: 12.8		
				Lignin: 12.8		
				Polysaccharides (starch + hemicellulose): 50.3		
				Starch: 45.0		
Peel	N/A	N/A	N/A	Cellulose: 39.0	N/A	75
				Hemicellulose: 25.0		
				Lignin: 8.0		
Peel	88.6	2.1	2.6	Fiber: 6.1	N/A	62
				Starch: 68.7		
Peel	97.4	3.2	13.0	12.0	N/A	76
Peel	86.0	5.3	3.7	Cellulose 14.2	N/A	74
				Hemicellulose 23.4		
				Lignin 10.8		
				Starch: 29.8		
Peel	20.0	N/A	4.2	Cellulose: 10.1	N/A	77
				Hemicellulose: 6.5		
				Lignin: 5.0		
Peel	90.2	N/A	6.4	45.6	N/A	78
Peel	30.0	0.1	1.3	Starch 23.2	N/A	79
				Amylose 16.4		
				Amylopectin 83.6		
Peel	30.1	3.5	7.0	Cellulose 14.0	N/A	80
				Hemicellulose 27.0		
				Lignin 11.0		
Peel	5.0	4.1	14.6	24.5	6.5 × 10^−3^	61
Peel	90.0	6.4	4.7	Cellulose 9.1	N/A	81
				Hemicellulose 7.5		
				Lignin 9.2		
				Starch: 44.5		
Peel	78.0	N/A	2.0	N/A	N/A	82
Peel (in natura)	13.7	6.2	4.9	10.9	118.9	83
Pulp	2.8	1.8	1.2	2.1	0.0	61
Root	88.3	2.4	2.1	10.3	N/A	62
Root	89.0	8.1	3.1	Polysaccharides: 11.0	40.0	84
				Lignin 16.0		
				Starch: 40.0		
Root	4.0	3.5	2.9	5.3	0.0	61
Stem	93.4	4.5	5.3	29.1	N/A	73
Stem	85.6	N/A	2.7	Cellulose: 39.9	N/A	58
				Hemicellulose: 11.7		
				Lignin: 22.6		
Stem	0.9	0.0	0.0	Cellulose 22.8	N/A	74
				Hemicellulose: 28.8		
				Lignin 22.1		
				Starch: 15.0		
Stem	1.2	5.2	6.4	39.5	7.0 × 10^−4^	61
Thick stalk	86.7	N/A	2.9	Cellulose: 40.7	N/A	58
				Hemicellulose: 12.1		
				Lignin: 20.0		
Thin stalk	85.2	N/A	2.8	Cellulose: 37.7	N/A	58
				Hemicellulose: 11.7		
				Lignin: 22.6		

a N/A = data not available.

These data highlight cassava leaves have the highest crude protein content among plant fractions, reaching 28.02%, plus high minerals (7.28% ash) and fiber (21.41%). These characteristics position them as a promising alternative for animal feed formulation, with an estimated energy value of 20.18 MJ kg^−1^ (Idris *et al.*, 2020). Tuber pulp shows carbohydrate predominance, low protein (1.75%), fiber (2.11%), and ash (1.24%), supporting its energy source role.^61,62^

The stem has the highest fiber content among the different parts of the plant, reaching 39.51%, accompanied by a moderate protein content (5.24%) and a mineral content of 6.43%. Discarded tubers present intermediate composition, with 3.54% crude protein, 2.94% ash, and 5.31% fiber, indicating potential for less nutritionally demanding applications.^61^

According to Veiga *et al.*,^58^ cassava root residues present lignocellulosic composition, with dry matter of 1.37–4.20% and ash contents of 2.5–6%. These values are influenced by washing procedures and soil contamination. Lignocellulosic characterisation shows cellulose predominance (33–44%), followed by extractives (17–33%), lignin (16–24%), and hemicellulose (10–14%). This composition is comparable to conventional woody biomass used in bioenergy applications. Higher heating values range from 16.3 to 18.9 MJ kg^−1^, highlighting potential for combustion and pyrolysis processes.

Cassava bagasse is the main solid byproduct of starch extraction, with moisture above 85% and dry matter between 12–35%, depending on pressing method.^59,60^ Bromatological analysis indicates high residual starch (60.8–64%), with crude protein 0.8–3.0%, ash 2.0–2.4%, and fiber 2.6–19.27%.^59^ Fiber and compositional variability depend on starch extraction efficiency.^68,70^ The cellulosic fraction reaches 19.27%, with low lignin (4.11%) and high residual starch (61.6%), favoring enzymatic hydrolysis and fermentation.^59,68^

SEM analysis shows cassava bagasse with abundant residual starch granules and fibrous structures typical of a cellulosic matrix.^65,68^After enzymatic treatment and high-pressure homogenisation, smoother fibers (10–15 µm) and nanofibrils (15–30 nm) are formed due to non-cellulosic removal.^68^

XRD analysis shows increased crystallinity after purification, from 20.9% to 63.4%, with α-amylose dihydrate as the main crystalline phase.^65,68^ Acid hydrolysis produces cellulose nanocrystals with high crystallinity (up to 54.1%) and high aspect ratio, suitable for polymer reinforcement.^64^

According to Elemike, Oseghale, and Okoye,^70^ garri processing residues contain high insoluble carbohydrates, reaching 76.6% dry matter, mainly residual starch and structural fibers. Their composition includes approximately 60.8% non-hydrolyzed starch, 15.8% fibers, low protein content (0.8%), and ash around 2.0%. This residual starch supports second-generation bioethanol production, achieving 32.4% yield after enzymatic saccharification and fermentation with *Saccharomyces cerevisiae*.

Cassava peel presents mineral-rich composition, with high volatiles (up to 81.98%), fixed carbon 11.83%, and ash between 2–10%, depending on variety and conditions.^76,82^ Elevated ash is linked to accumulated inorganic minerals, such as calcium, magnesium, and potassium, and possible soil contamination during harvesting and processing.^83^ Mineral analysis shows high potassium (up to 8570 mg kg^−1^), calcium (5300 mg kg^−1^), and crude fiber up to 24.49%, exceeding levels found in bagasse.^61,65,76^

Elemental analysis indicates predominance of carbon (43%) and oxygen (46%), with minor nitrogen fractions, suggesting the presence of residual protein compounds.^65^ During hydrothermal gasification, cassava peel shows typical values of 76% moisture, 22% volatiles, and 2.0% ash, affecting energy conversion efficiency.^82^ Its mineral-rich composition enables heavy metal adsorption, reaching 42.46 mg g^−1^ for Pb^2+^ and 43.97 mg g^−1^ for Cr^3+^ after chemical modification.^65,85^

According to Fanelli *et al.*,^62^ residue disposal methods influence quality and bromatological properties, affecting dry matter, mineral content, and fiber composition. In their study, peeled and unpeeled cassava roots used as feed showed similar dry matter contents (88%) and low crude protein levels (2.0–2.1%). Peel presence increased ash (2.6–3.0%) and total fiber contents (6–10%), including higher insoluble (5–8%) and soluble fiber fractions (1–2%). Peeling reduced mineral and fiber contents while maintaining energy value, indicating both forms are nutritionally suitable for animal feed with distinct profiles. Cassava wastewater, generated during pulp pressing, was excluded from Table 1 due to its liquid nature and distinct chemical composition.

According to Oliveira *et al.*,^87^ these effluent exhibits acidic pH (3.9), high total solids (92.9 g L^−1^), and volatile solids (73.4 g L^−1^), indicating high organic content. High biochemical (29.2 g O_2_ per L) and chemical oxygen demand (101.38 g O_2_ per L) confirm its elevated organic load and bioconversion potential. Elemental composition includes 33.40% carbon, 7.12% hydrogen, and 2.14% nitrogen, resulting in a C/N ratio of 16.66, suitable for anaerobic digestion. High native microbial populations, including bacteria and yeasts (10^8^ CFU mL^−1^), enhance fermentative capacity for biogas production.

Thermogravimetric analysis indicates moderate thermal stability of cassava residues, with major degradation between 235–325 °C due to cellulose and hemicellulose decomposition.^61,88^ Carbonisation residues range from 20–25% at 550 °C, reflecting the formation of thermally stable char fractions.^65^ Chemical or enzymatic treatments enhance thermal stability, shifting peak decomposition temperatures to above 325 °C through structural modification of cellulosic components. Lignin and extractives contribute to higher thermal resistance above 320 °C, expanding the potential for thermochemical valorisation.^68,89^ Under slow pyrolysis at 500–600 °C, cassava residues yield biochar with high carbon content (up to 81.35%) and large surface area. The resulting biochar presents suitable pore volume for water and nutrient retention, supporting its application as a soil amendment.^86^

Cassava residues contain cyanogenic compounds, mainly linamarin and lotaustralin, whose concentrations depend on variety, edaphoclimatic conditions, and processing methods.^90^ Cyanogenic levels vary widely among plant parts, ranging from trace amounts in stems to higher concentrations in leaves and tubers. Reported concentrations include 7.03 × 10^−4^ mg kg^−1^ in stems, 0.01 mg kg^−1^ in tubers, and 0.02 mg kg^−1^ in leaves. Despite variability, all measured values remain below internationally established safety thresholds.^61^ Traditional processing methods reduce cyanogenic compounds but may not consistently achieve the WHO safety limit of 10 ppm. This limitation is intensified during drought periods, when cyanide accumulation in cassava roots increases significantly.^90^ Elevated cyanide levels raise risks of acute poisoning and chronic neurological diseases, including konzo and tropical ataxic neuropathy. Intensive washing, controlled drying, and mechanical grinding efficiently reduce cyanogenic compounds in feed-grade cassava roots.^62,65^ These treatments ensure safety for animal feed and expand the potential of cassava residues for industrial and food-related applications.

**Fig. 3:**
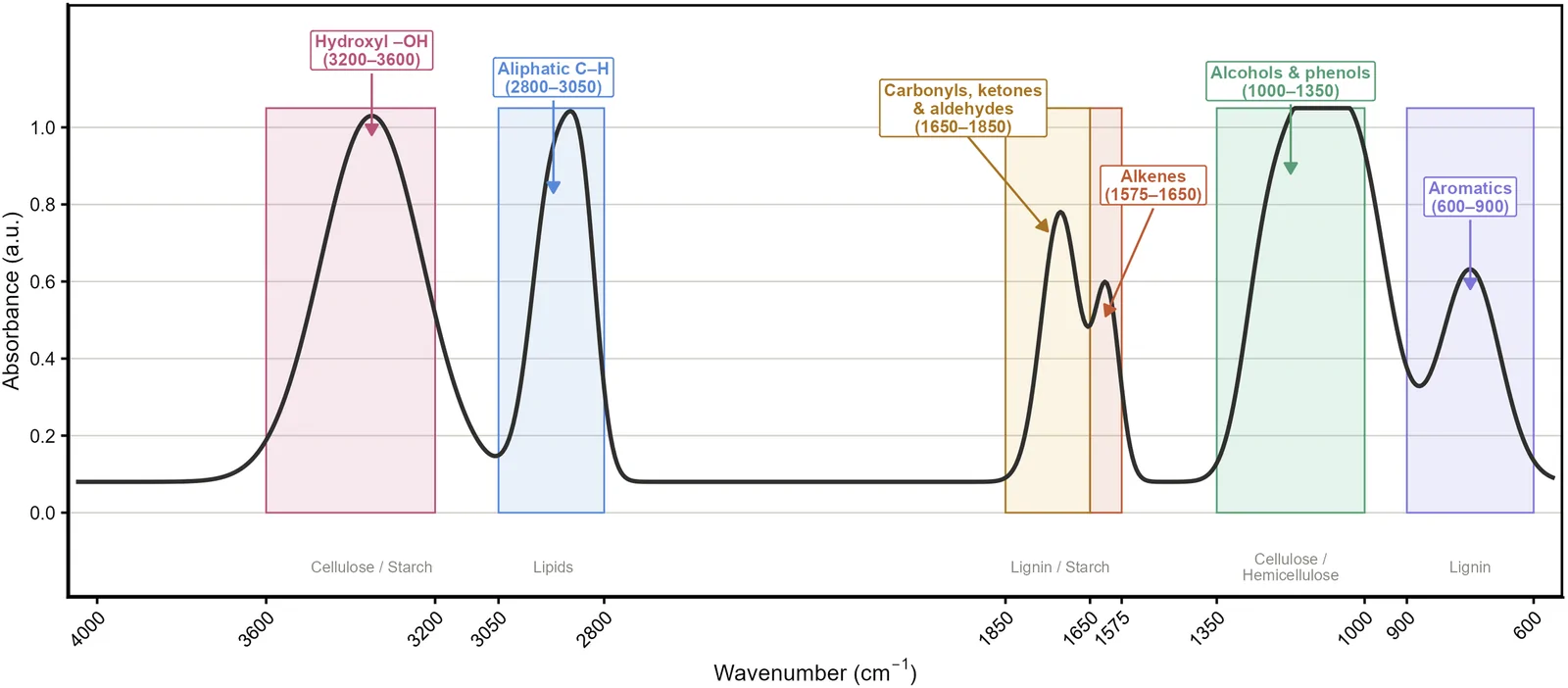
Schematic FTIR spectrum of cassava residues showing the main absorption bands and associated functional groups. Coloured regions indicate the wavenumber range of each group; structural components linked to each band are indicated below the *x*-axis. Prepared by the author based on Williams & Onwudili,^82^ Idris *et al.*,^61^ Thuppahige *et al.*,^65^ Hazrati *et al.*^88^

FTIR analysis reveals functional groups typical of both lignocellulosic and starchy materials in cassava residues (Fig. 3). Broad hydroxyl bands (3200–3600 cm^−1^) and aliphatic C–H stretching (2800–3050 cm^−1^) indicate cellulose, hemicellulose, and starch abundance. Carbonyl absorptions (1650–1850 cm^−1^) and aromatic signals (600–900 cm^−1^) reflect contributions from lignin and phenolic compounds.^82^ This chemical heterogeneity supports multiple valorisation routes, including thermochemical conversion and applications in polymeric and biodegradable composites.^68^

The compositional diversity of cassava residues, shown by the bromatological data in Table 1 and structural analyses, reflects genetic variability, cultivation conditions, and processing practices. Processing methods strongly influence starch extraction, fiber removal, and the concentration of minor components such as proteins, lipids, and minerals.^58–60,62^ This heterogeneity gives cassava residues a broad range of applications, including biofuels, biomaterials, animal feed, adsorbents, and soil amendments.^64,85,86^ Applications include bioethanol, biogas, biochar, nanocellulose, biodegradable films, composites, active packaging, and materials for heavy-metal removal.^89,91^

This diversity highlights the need for careful and systematic characterisation to define appropriate technological routes for agro-industrial and environmental valorisation.^58,87^Such approaches contribute to the sustainability of the cassava production chain and promote circular economy principles in the agricultural sector.^91^

## 4. Trends in cassava waste valorisation

**Fig. 4:**
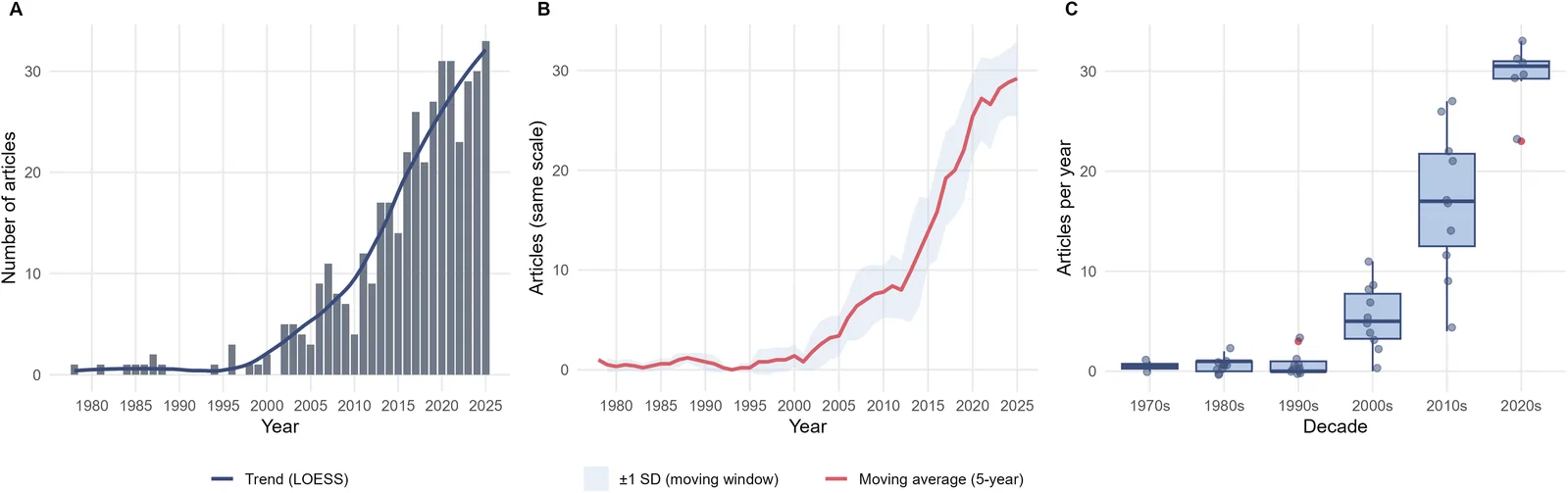
Temporal evolution of publications on cassava waste (1978–2025). (A) Number of articles per year with LOESS trend curve. (B) 5 year moving average with variability range (±1 standard deviation). (C) Distribution of annual publications by decade. Source: Authors' elaboration based on data retrieved from the Scopus database (accessed on 19 January 2025).

To evaluate publication trends on cassava residues, a search was conducted in the Scopus database (Elsevier). The database was accessed on January 19, 2025. The search string used was “cassava residues” OR “cassava waste”. These terms are widely adopted to describe by-products from cassava processing. The results were limited to articles and reviews published between 1978 and 2025. A total of 414 studies were identified. The retrieved data were used to construct Fig. 4.

Temporal analysis shows a clear evolution of scientific interest over recent decades, indicating the progressive consolidation of research on cassava residues. Between the 1970s and 1990s, annual publication output was low and irregular, with medians near zero and limited dispersion (Fig. 4A and C). This pattern characterizes an emerging field marked by sporadic, unsystematic research activity. During this period, cassava waste remained peripheral to major scientific agendas, mainly discussed as an environmental liability from flour and starch processing. Early literature focused on describing waste streams and assessing environmental impacts of improper disposal, with limited emphasis on technological valorisation.^92^ Cassava residues were predominantly regarded as unwanted agro-industrial byproducts rather than potential resources in this initial research phase.

From the 2000s onward, publications increased steadily, as shown by the LOESS smoothing curve, indicating sustained growth in research activity (Fig. 4A). This period marks a shift from predominantly environmental assessments to a technological perspective recognizing cassava residues as carbohydrate-rich substrates. Such repositioning is linked to advances in fermentation, anaerobic digestion, and biorefinery concepts for integrated cassava residue utilization.^11,92^

After 2010, the topic gained visibility within agro-industrial waste valorisation, bioenergy, and circular economy research. The moving average (±1 standard deviation) indicates early instability followed by increased output and reduced variability, evidencing field maturity (Fig. 4B). At this stage, literature increasingly positions cassava waste within bioeconomy and biorefinery frameworks. Studies demonstrate viable integrated routes for producing biogas, bioethanol, biohydrogen, enzymes, and organic acids, strengthening technological and economic relevance.^11,19,93^

The decadal boxplot shows higher medians and greater variability in recent decades, indicating increased publication output and diversified research approaches (Fig. 4C). This advancement is closely linked to circular economy principles promoting waste reduction and reintegration of residual flows into production chains. Consequently, cassava waste shifts from low-value byproducts or environmental liabilities to strategic raw materials in integrated, sustainable production systems.^93,94^

Methodological deepening and diversification of experimental approaches include more efficient physical, chemical, and biological pretreatments, along with hybrid and integrated processes. Recent reviews and experimental studies demonstrate this maturity by exploring optimized routes for converting cassava waste into bioenergy and higher value-added bioproducts.^46,95^

Taken together, the data reveal a transition from environmentally focused studies to a consolidated field recognizing cassava waste as a strategic resource for bio-based applications.^11,94^

## 5. Methods for recovering cassava waste

Analysis of cassava production and publication trends reveals large volumes of residues and increasing recognition of their biotechnological and thermochemical potential, driving residue valorisation strategies. These approaches vary in operational complexity, mechanisms of action, and process integration, reflecting residue heterogeneity and targeted products.^74,78^

**Fig. 5:**
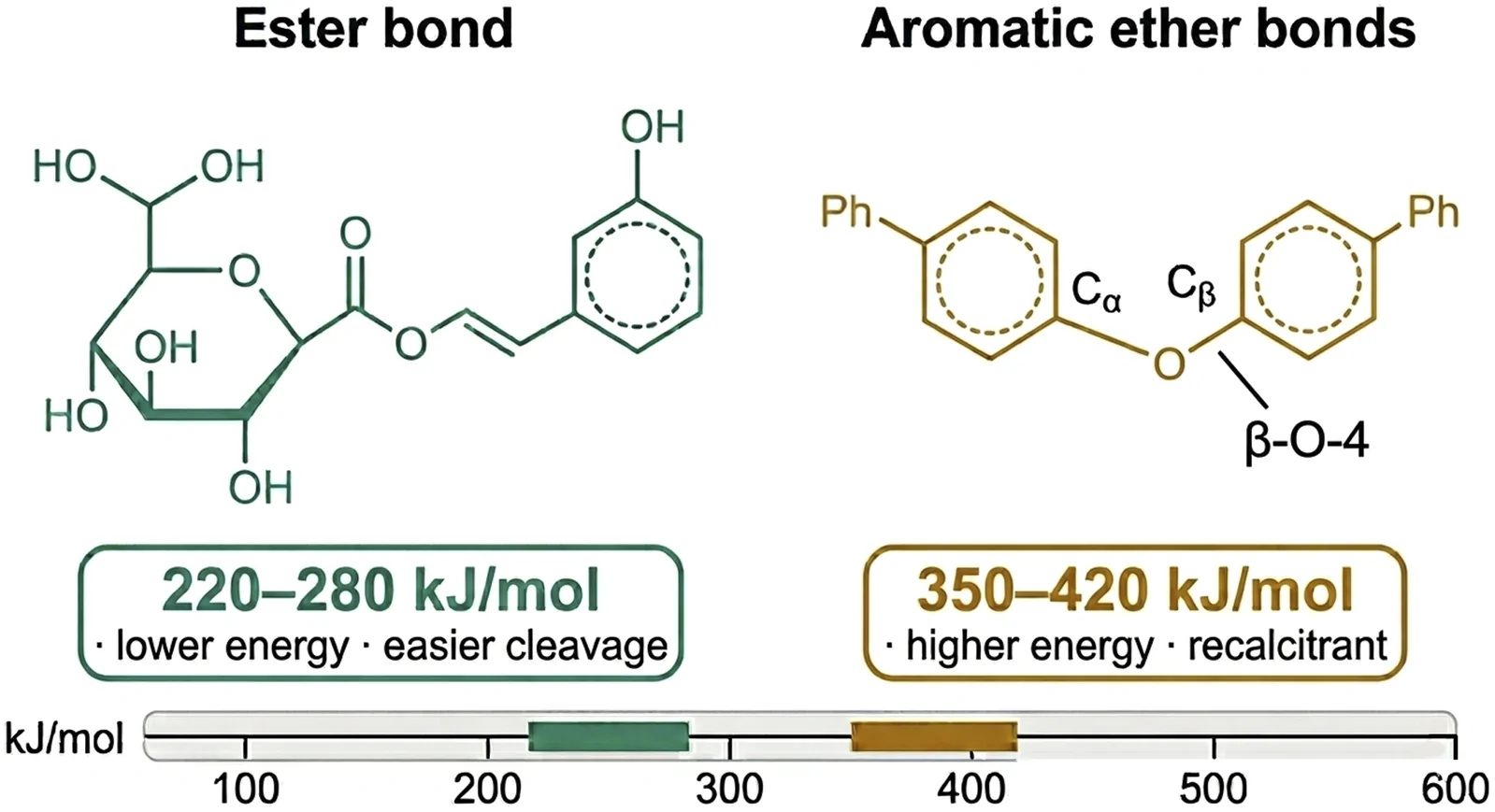
Schematic representation of the main bond types governing the thermodynamic recalcitrance of lignocellulosic biomass. Prepared by the author based on Mosier *et al.*^96^ and Agbor *et al.*^98^

The lignocellulosic matrix exhibits structural recalcitrance due to covalent and non-covalent interactions among lignin, hemicellulose, and cellulose, limiting enzymatic and microbial accessibility. Ester bonds show dissociation energies of 220–280 kJ mol^−1^, whereas lignin ether linkages require 350–420 kJ mol^−1^, reflecting aromatic electron delocalization (Fig. 5).^96–98^ This energetic disparity explains why mild treatments disrupt ester bonds, while lignin depolymerisation requires harsher thermochemical conditions or oxidative catalysts.^99^

From a kinetic perspective, polysaccharide hydrolysis is governed by the activation energy of glycosidic bond cleavage reactions. In dilute acidic media, hemicellulose hydrolysis shows activation energies of 100–120 kJ mol^−1^, whereas crystalline cellulose requires 180–220 kJ mol^−1^ due to chain packing and hydrogen bonding.^100^ Therefore, pretreatments reduce energy barriers by structural disorganization, increased surface accessibility, and surface chemistry modification, requiring condition adjustments according to residue fraction variability (Table 1).

**Fig. 6:**
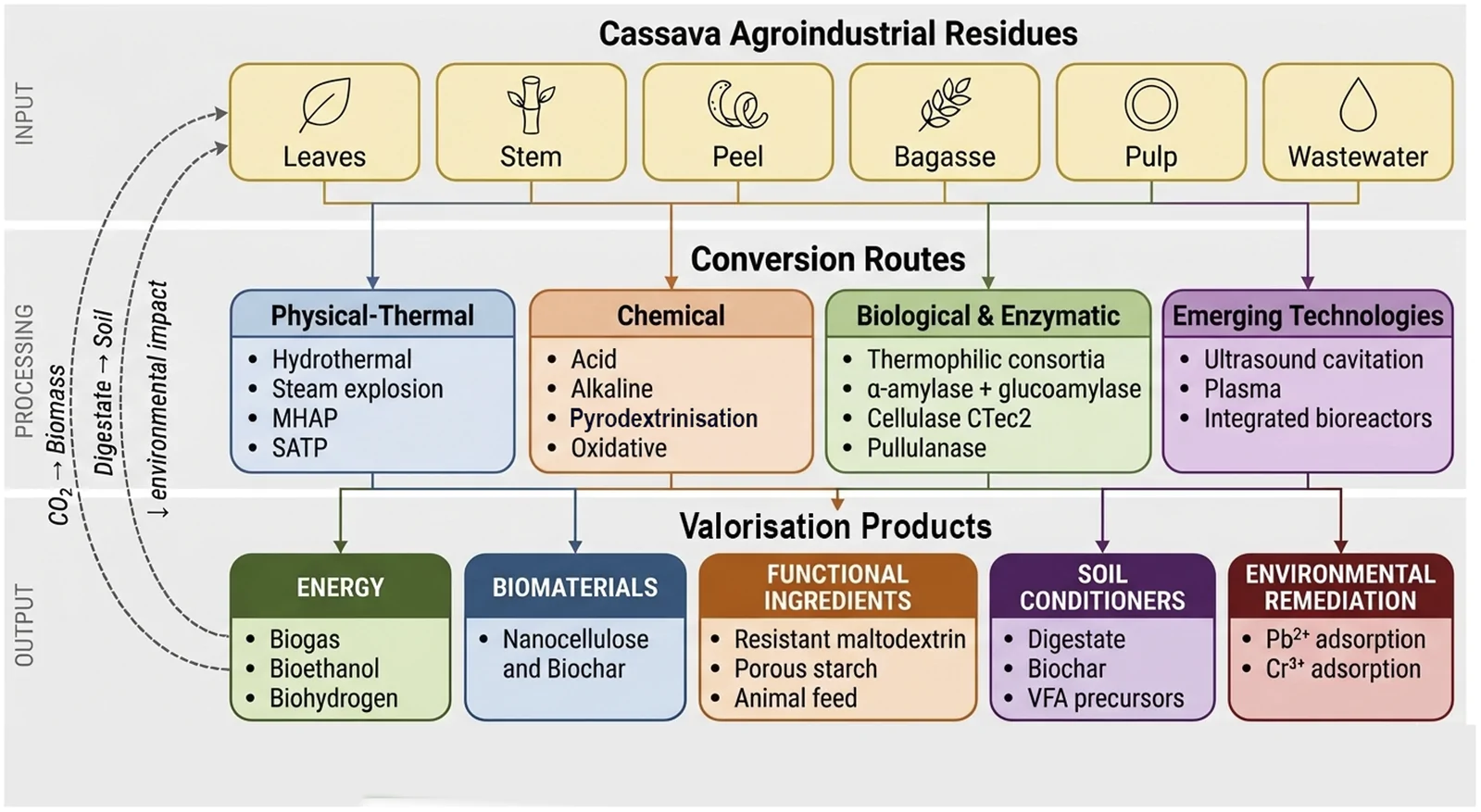
Integrated model of a circular biorefinery based on cassava production chain waste, illustrating the main residual fractions generated, the available conversion routes, and the products and by-products obtained. Prepared by the author based on Idris *et al.*,^61^ Veiga *et al.*,^58^ Fanelli *et al.*,^62^ Oliveira *et al.*,^87^ Pooja e Padmaja,^74^ Varongchayakul *et al.*,^101^ Zhang *et al.*,^72^Moshi *et al.*,^84^ Aruwajoye, Faloye and Kana,^81^ Nitayavardhana *et al.*,^102^ Hu *et al.*,^103^ Panyasiri *et al.*,^68^ Pasquini *et al.*,^64^ Hierro-Iglesias *et al.*,^86^ Wijitkosum e Jiwnok,^86,104^ Pattarapanawan *et al.*,^104^ Gonzalez *et al.*,^105^ Chen *et al.*,^106^ Meier *et al.*,^107^ Fasheun *et al.*,^108^ Peres *et al.*,^109^ Chogi *et al.*,^109^ Schwantes *et al.*,^85^ Carvalho *et al.*^110^ e Agudelo-Patiño *et al.*^111^

Fig. 6 presents an integrated circular biorefinery model for cassava chain residues, outlining waste fractions, conversion routes, and derived products, discussed in subsequent sections.

### 5.1. Physical and physico-thermal pretreatments

#### 5.1.1. Hydrothermal treatment

Hydrothermal pretreatment is widely used for lignocellulosic biomass due to operational simplicity, absence of chemicals, and efficiency in plant matrix modification. Exposure to saturated steam at 120–180 °C causes cell wall swelling and partial hemicellulose solubilisation^112,113^(Chen *et al.*, 2017; Fan *et al.*, 2025). The process also promotes cleavage of ester and ether bonds linking lignin and polysaccharides, typical of autohydrolysis and hydrothermal pretreatments.^112,113^

High temperature and pressurized water promote hemicellulose autohydrolysis, catalyzed by organic acids released during thermal treatment. Acetic acid, generated from hemicellulose acetyl group dissociation, plays a key catalytic role in hydrothermal pretreatment mechanisms.^112^ Higher temperatures accelerate β-1,4-glycosidic bond cleavage in xylans and arabinans, forming soluble oligosaccharides transferred to the liquid phase. Structural effects include cell wall thinning, cell separation, and partial lignin depolymerisation *via* α-O-4 and β-O-4 cleavage, increasing lignin mobility.^114,115^

In cassava peels, high starch content adds complexity to pretreatment, as starch contributes structurally and functionally to the lignocellulosic matrix. Starch gelatinisation disrupts its semicrystalline structure, increasing molecular mobility, hydrodynamic expansion, and matrix destabilisation. During hydrothermal treatment, these changes enhance solubility, swelling, and enzymatic reactivity, facilitating subsequent amylase and cellulase action.^81^

Cassava peel starch shows gelatinisation between 65–85 °C, with maximum swelling at 65–75 °C, as reported by Fronza *et al.*^79^ This behavior is driven by high amylopectin content (83.6%), whose branched structure reduces disruption energy and accelerates crystalline disorganisation. Pre-gelatinisation before thermocompression increases density and mechanical strength of starch biocomposites, although reducing expansion capacity.^116^ Overall, gelatinisation kinetics and thermodynamics modulate mechanical, thermal, and rheological properties, underpinning cassava waste valorisation in biodegradable polymer matrices.

Experimental results confirm combined effects of starch gelatinisation and lignocellulosic matrix disruption during cassava peel pretreatments. Steam treatment at 120 °C for 30 min causes matrix swelling, hemicellulose rupture, lignin solubilisation, and starch gelatinisation.^74^ These physicochemical changes increase biomass porosity and accessible surface area, enhancing cellulolytic enzyme efficiency. Reducing sugar production reached 57.6 g per 100 g of peels, exceeding yields from longer treatments and alternative pretreatments. Lower yields were observed after prolonged steam exposure and microwave-assisted acidic or dilute acid pretreatments. These results highlight the importance of controlling gelatinisation mechanisms to optimize biochemical conversion of cassava residues. Despite advantages, severe hydrothermal conditions may generate inhibitory compounds affecting downstream bioconversion. Pentose and hexose degradation forms furfural and HMF, while partial lignin decomposition releases phenolic compounds.^117^ These inhibitors reduce enzymatic activity and fermentative yeast viability, decreasing bioethanol and biogas yields. Therefore, pretreatment parameters must be optimized for each biomass to maximize polysaccharide solubilisation and minimize inhibitor formation.

#### 5.1.2. Soaking Assisted Thermal Pretreatment (SATP)

Soaking Assisted Thermal Pretreatment (SATP) is a refined hydrothermal variant combining dilute acid soaking with subsequent autoclaving. Only one Scopus-indexed study was identified using the terms “SATP” and “biomass”, specifically addressing cassava residues. Aruwajoye, Faloye, and Kana^81^ applied SATP to cassava peels and optimized conditions using response surface methodology. Optimal conditions included 69.62 °C soaking, 2.57 h immersion, 3.68% HCl, autoclaving at 121 °C for 5 min, and 9.65% solids. Under these conditions, reducing sugars reached 89.80 ± 2.87 g L^−1^, corresponding to 0.93 g g^−1^ peel and 90.79% recovery. A combined severity factor of 0.77 indicated low inhibitor formation, favoring subsequent fermentation processes. According to the authors, SATP achieved a 31% improvement compared to isolated enzymatic pretreatment.

Aruwajoye, Faloye, and Kana^81^ described SATP as a two-step synergistic pretreatment mechanism. First, immersion in dilute acid promotes partial hemicellulose hydrolysis and activates endogenous acid groups in the biomass. Second, autoclaving enhances depolymerisation through combined temperature and pressure effects, without requiring high mineral acid concentrations. Controlled hemicellulose solubilisation and partial lignin degradation increase matrix porosity. These changes expose crystalline cellulose, improving its accessibility to enzymatic hydrolysis.

#### 5.1.3. Microwave and steam assisted pretreatments

Comparisons between microwave-assisted (MHAP) and steam-assisted acid pretreatments (SHAP) reveal significant structural and energetic differences in cassava residues. Cheng *et al.*^71^ evaluated both methods for cassava waste targeting hydrogen and methane cogeneration. MHAP formed regular micropores (6 µm) and higher crystallinity (33.00), whereas SHAP produced irregular fragments (23 µm) and wider fissures (0.2 µm). After enzymatic hydrolysis, reducing sugars reached 38.9 g/100 g for MHAP and 43.6 g/100 g for SHAP. These yields corresponded to 78.6% and 88.2% of theoretical conversion for MHAP and SHAP, respectively. During fermentation, MHAP favored hydrogen production (106.2 mL per g TVS), while SHAP enhanced methane generation (93.2 mL per g TVS). Overall energy conversion efficiency increased to 24.7%, reflecting pretreatment-dependent pathway selection.

Microwave heating relies on electromagnetic absorption by polar molecules, promoting rapid and uniform volumetric heating of lignocellulosic biomass. This mechanism induces localized glycosidic bond rupture and micropore formation, increasing biomass surface area and reducing cellulose crystallinity.^118,119^ Steam-assisted acid pretreatment operates at high temperature and pressure, followed by sudden decompression typical of steam explosion. This explosive decompression expands the cellular matrix, generating fissures, porosity, and mechanical fragmentation, enhancing enzymatic accessibility.^120,121^ High thermal severity intensifies catalytic autohydrolysis, promoting hemicellulose degradation and oligosaccharide release. The synergy between acid hydrolysis and physical fragmentation explains higher reducing sugar yields in SHAP, such as 43.6 g/100 g reported in similar studies.^120,122^

Both pretreatments risk forming inhibitors such as furfural, HMF, phenolics, and organic acids under severe operating conditions. Methane yields of 75–93 mL per g TVS were reported, substantially lower than typical values for cassava residues (249–350 mL g^−1^ TVS), indicating inhibition.^71^ Reduced yields were attributed to inhibitory compounds generated during pretreatment, negatively affecting methanogenic activity. These findings highlight the need for detoxification strategies, including washing, activated carbon adsorption, or enzymatic treatment with laccases. Optimization of operating conditions is also required to limit sugar degradation and minimize inhibitor formation.

Industrial application of microwave heating faces technical and economic limitations, restricting its feasibility in large-scale continuous biomass pretreatment processes. High energy demand arises from limited microwave penetration depth and the need for high power, particularly when processing moist materials.^123^ Microwave pretreatment requires specialized reactors to ensure homogeneous electromagnetic fields and prevent hotspots, increasing operational complexity.^123,124^ Capital costs are elevated due to high-power generators, custom cavities, and dielectric-compatible construction materials.^123,125^ Consequently, microwave systems are generally more expensive than conventional autoclaves and thermal equipment for industrial-scale applications. In contrast, steam-assisted pretreatment shows greater technological maturity and has been validated at pilot and industrial scales. SHAP offers robust performance, operational simplicity, and comparatively lower investment and operating costs. Steam explosion is recognized as a well-established biomass pretreatment technology, combining efficiency, low energy demand, and large-scale applicability.^120,126^

**Fig. 7:**
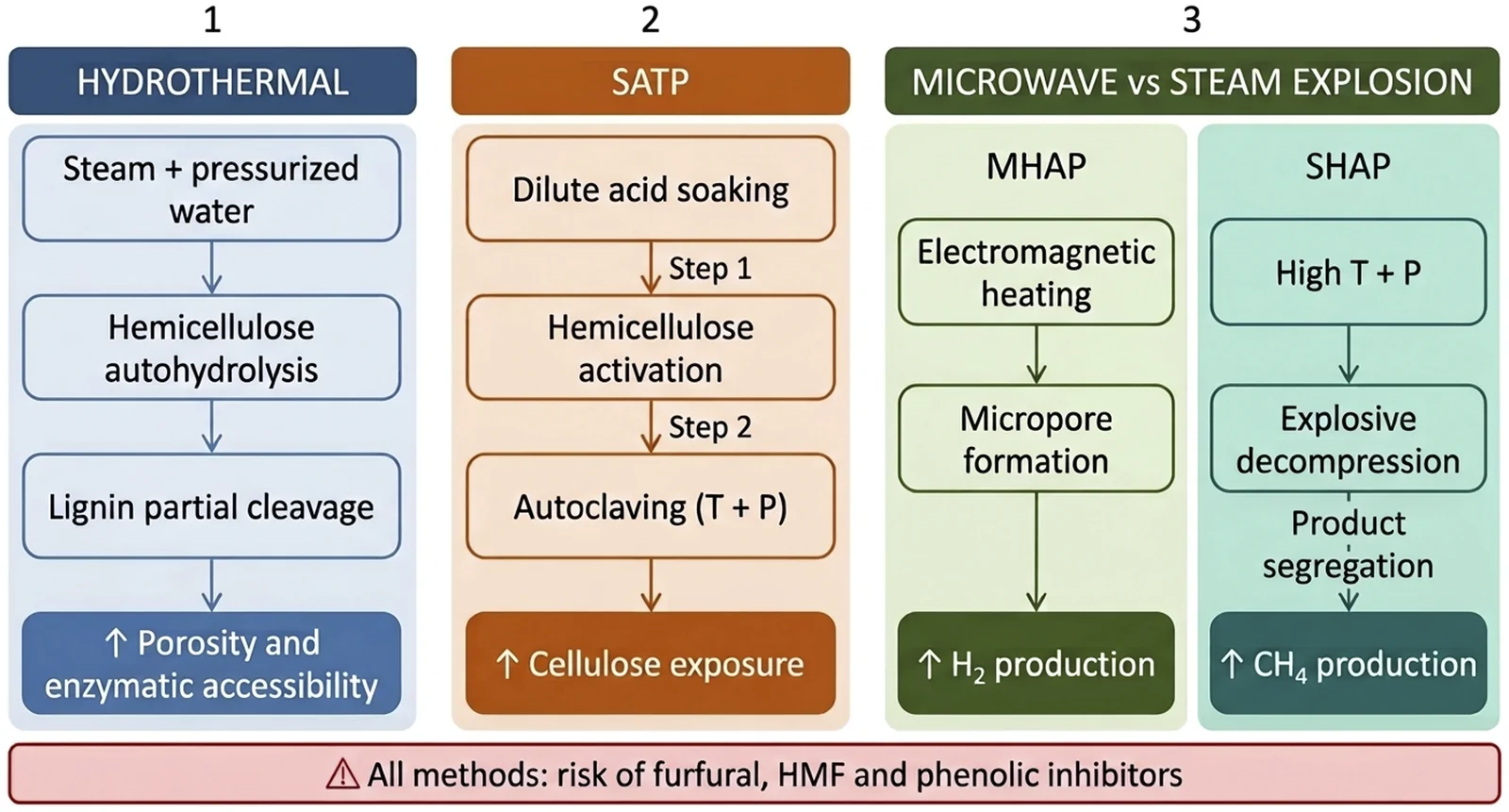
Schematic flowchart of hydrothermal, SATP, MHAP, and SHAP pretreatments applied to cassava lignocellulosic residues, highlighting key mechanisms and dominant reaction pathways. Prepared by the author based on Chen *et al.*,^112^ Pooja and Padmaja,^74^ Aruwajoye, Faloye and Kana,^81^ Cheng *et al.*,^71^ Ziegler-Devin *et al.*,^120^ Venegas-Vásconez *et al.*^118^ e Ahmed *et al.*^117^

Fig. 7 summarizes the mechanistic flow of the three methods discussed. Although distinct in operational principle, these method converge on the same objective: to increase the enzymatic accessibility of biomass.

### 5.2. Acid pretreatment

Acid pretreatment is widely applied to lignocellulosic biomass because it selectively removes hemicellulose and increases plant matrix porosity.^127^ Due to lower crystallinity and polymerisation degree, hemicellulose is more susceptible to hydrolysis than cellulose under acidic conditions. The mechanism involves protonation of β-1,4-glycosidic bonds, followed by heterolytic cleavage, releasing monosaccharides and soluble oligosaccharides.^96^

Acid pretreatment not only acts on hemicellulose but also promotes additional structural changes in biomass. These include partial lignin breakdown and cell wall disorganisation, which together enhance enzymatic accessibility in later conversion stages.

The kinetics of acid hydrolysis of lignocellulosic biomass are described by first-order models with respect to the polymeric substrate.^128^ Several studies report biphasic behavior, comprising a fast-reacting fraction and a slow-reacting fraction, associated with structural heterogeneities of the material.^129^ The fast phase is attributed to hemicellulose solubilisation and amorphous regions, exhibiting higher first-order rate constants, defined as *k*_1_.^130^ At temperatures between 155 and 185 °C, *k*_1_ is approximately one order of magnitude higher than the constant of the slow fraction.^129^ The slow phase is mainly associated with crystalline cellulose hydrolysis, whose limited accessibility results in lower rate constants, represented as *k*_2_.^131,132^ In a simplified way, the decomposition of hemicellulose can be expressed as eqn (1):

1 (C_5_H_8_O_4_)_*n*_ + *n*H_2_O → *n*C_5_H_10_O_5_

The efficiency of acid pretreatment depends on balancing polysaccharide solubilisation with minimisation of inhibitory compound formation. This balance was demonstrated by Zhang *et al.*^133^ (2011) through optimisation of acid thermal pretreatment of cassava waste using RSM and CCD. Optimal conditions of 157.84 °C, 2.99% H_2_SO_4_, and 20.15 min achieved a maximum methane yield of 248 mL CH_4_ per g VS. This represented a 56.96% increase compared to the control yield of 158 mL CH_4_ per g VS. Enhanced performance was attributed to hemicellulose solubilisation and partial lignin degradation, increasing porosity and improving anaerobic digestion efficiency.

However, the same study demonstrated that excessively severe conditions (temperatures above 170 °C, acid concentrations above 4%, and times exceeding 30 minutes) favor the formation of furfural and HMF, compromising biomethanogenic yield. To predict this balance between severity and performance, the severity factor (SF) is used, expressed in eqn (2):2
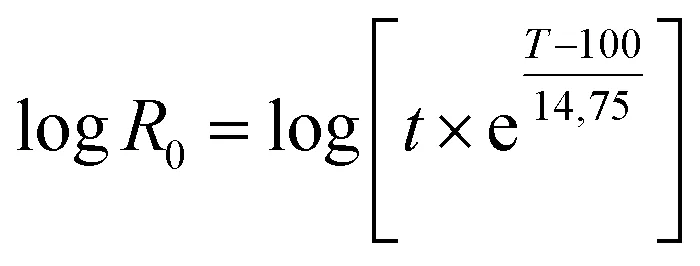


The severity factor (log *R*_0_) integrates time and temperature effects, quantifying hydrothermal pretreatment intensity; values of 3.5–4.0 enable efficient hydrolysis with minimal inhibitors.^133^

Awoyale and Lokhat^78^ (2021) demonstrated the effectiveness of acid pretreatment using Nigerian lignocellulosic biomasses under hydrothermal, hydrothermal-acid, and hydrothermal-alkaline conditions. Hydrothermal-acid pretreatment increased cassava peel cellulose from 25.8% to 43.8%, accompanied by a significant reduction in lignin content. FTIR, SEM, BET, and XRD analyses confirmed partial conversion of crystalline cellulose into amorphous form, with increased surface area and pore volume. Bioethanol production from acid-pretreated cassava and yam peels reached 0.41 USD per L, competitive with Nigeria's average market price of 0.45 USD per L. These findings demonstrate that pretreatment efficiency and reagent costs are decisive factors for the commercial viability of biofuels from lignocellulosic residues.

One major challenge of acid pretreatments is the formation of inhibitory compounds, similarly to severe thermal processes.^113^ The use of mineral acids under harsh conditions also accelerates equipment corrosion, increasing capital and maintenance costs.^134^ Severe acidic and thermal conditions promote pentose and hexose degradation into furfural and HMF, causing significant sugar losses.^113,135^ Acid pretreatment requires large reagent volumes followed by neutralisation and detoxification, generating saline and toxic effluents with high environmental impact.^135,136^ The low selectivity of acids may lead to excessive lignin solubilisation and cellulose degradation, reducing process efficiency.^137^ Additional detoxification steps, such as activated carbon or oxidative treatments, further increase operational complexity.^113,138^ Consequently, these limitations restrict acid pretreatment integration into sustainable biorefineries focused on low environmental impact and circular resource use.

### 5.3. Alkaline pretreatment

Alkaline pretreatment, particularly using sodium hydroxide (NaOH), is effective for delignification and improving enzymatic accessibility in lignocellulosic biomass.^139^ Its action differs from acid treatment and involves complementary chemical mechanisms. Ester bond cleavage occurs through saponification, wherein acetyl groups at the C-2 and C-3 positions of hemicellulosic xylose residues are hydrolyzed. In alkaline media, this reaction forms sodium acetate and releases free hydroxyl groups, increasing fiber reactivity.^100^ This deacetylation reduces steric hindrance and increases the hydrophilicity of hemicellulose, facilitating its solubilisation and subsequent enzymatic hydrolysis.

The alkaline depolymerisation of polysaccharides proceeds mainly through peeling reactions at the reducing ends of polymer chains. Under alkaline conditions, the terminal C-1 carbon undergoes β-eliminative rearrangement, forming saccharinic acid derivatives that detach from the main chain.^140^ This end-wise reaction leads to a progressive decrease in the degree of polymerisation during alkaline treatment.^141^ The peeling reaction is temperature-dependent and follows pseudo-first-order kinetics.^140,142^ Peeling competes with stopping reactions, in which reducing end groups are converted into stable carboxylic acid derivatives, limiting further depolymerisation.^141,143^

Alkaline delignification proceeds through cleavage of aryl ether bonds in lignin, particularly the β-O-4 linkage. Deprotonation of the α-hydroxyl group promotes a quinone methide intermediate, which undergoes nucleophilic attack releasing alkali-soluble lignin fragments.^143^

Salihu *et al.*^75^ evaluated eleven agricultural residues, including cassava peels, as substrates to produce cellulolytic enzymes (CMCase, FPase, and β-glucosidase) by *Aspergillus niger* in solid-state fermentation. The residues were subjected to three types of pretreatments: acid (H_2_SO_4_), oxidative (H_2_O_2_), and alkaline (NaOH). The alkaline pretreatment promoted significant delignification, increasing the porosity of the structure and exposing the cellulose, which resulted in greater enzymatic accessibility. The activities of the cellulolytic enzymes produced were substantially higher (up to 3.2 times higher) compared to the acid and oxidative pretreatments. Hence the alkaline treatment was the most effective in enhancing the use of cassava peels as a cheap and abundant substrate to produce enzymes of industrial interest.

A critical structural effect of alkaline treatment is the conversion of cellulose I (native) to cellulose II (mercerized), which occurs when the NaOH concentration exceeds 8–12% (w/v).^144,145^ Mechanistically, OH^−^ and Na^+^ ions penetrate between the cellulose chains, breaking intramolecular (O3–H⋯O5′) and intermolecular (O6–H⋯O2′) hydrogen bonds. This allows the chains to rearrange into a thermodynamically more stable antiparallel conformation. Cellulose II exhibits lower crystallinity, with crystallinity index reducing from 60–70% to 50–60%). In addition, this leads greater spacing between crystalline planes (from 0.39 nm to 0.44 nm), and greater accessibility to the cellulose-binding domains (CBM) of cellulases. Consequently, hydrolysis rates 30–80% higher compared to native cellulose I are obatined.^144^

Moshi *et al.*^84^ investigated the use of wild cassava (*Manihot glaziovii*) waste for combined bioethanol and biogas production. Alkaline pretreatment followed by enzymatic hydrolysis promoted higher biomass solubilisation, achieving ethanol yields up to 95% of the theoretical value. Volumetric ethanol productivity reached 1.2–1.3 g L^−1^ h^−1^, compared to only 0.2–0.5 g L^−1^ h^−1^ obtained using enzymes alone. The same pretreatment increased methane yields by up to 56%, reaching 316–352 L kg^−1^ of added volatile solids. Combined ethanol and methane production increased total energy output by 3–4 times over ethanol alone and 1.2–1.3 times over biogas alone.

NaOH pretreatment cleaves ester and partial ether bonds between lignin and hemicellulose, promoting lignin solubilisation in alkaline media.^146,147^ Hemicellulose undergoes partial degradation and solubilisation, releasing sugars such as xylose and arabinose and acidic derivatives.^147^ Alkaline conditions reduce cellulose crystallinity *via* cellulose I–to–cellulose II conversion, known as mercerisation, occurring above 10% NaOH.^145^ These chemical changes increase porosity and surface area through fiber swelling, microfibril disorganisation, and removal of amorphous components.^148^ Alkaline treatment also modifies biomass surface charge, enhancing electrostatic interactions between enzymes and cellulose and improving enzymatic accessibility.^149,150^

Alkaline pretreatment generates dark effluents with high pH (>12) and elevated organic load, including soluble lignin, phenolic compounds, and organic acids. Effluent neutralisation requires mineral acids, producing inorganic salts that complicate treatment and final disposal.^150^ Phenolic compounds derived from lignin strongly inhibit hydrolytic enzymes and fermentative microorganisms, reducing downstream conversion efficiency. Mitigation strategies include biomass washing, adsorption processes, resin application, and enzymatic oxidative treatments using laccases or peroxidases.^113^

### 5.4. Biological and enzymatic pretreatments

**Fig. 8:**
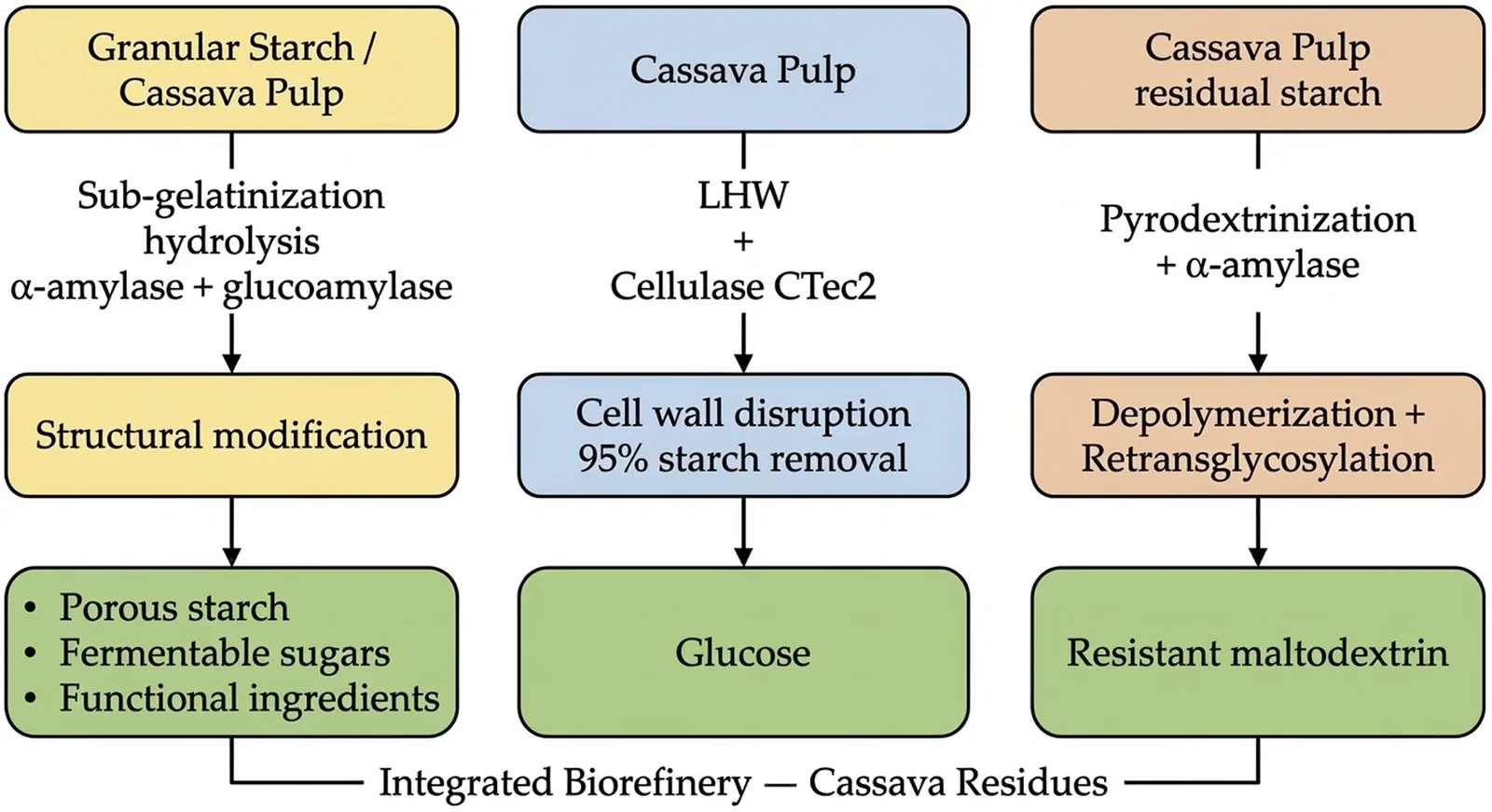
Enzymatic hydrolysis routes applied to the main residues of the cassava production chain and their respective valorisation products. Prepared by the author based on Uthumporn *et al.*,^106,151^ Chen *et al.*,^106^ Varongchayakul *et al.*,^152^ Pattarapanawan *et al.*^104^ e Gonzalez *et al.*^105^

The biotechnological valorisation of cassava residues relies on biological and enzymatic approaches. These methods vary in function depending on the substrate, target product, and processing stage. Unlike physicochemical methods, they operate under mild conditions. They generate fewer inhibitors and are more compatible with subsequent fermentation or anaerobic digestion steps. Two functionally distinct roles must be distinguished. The first is biological or enzymatic pretreatment, which prepares the biomass for further conversion. The second is enzymatic hydrolysis itself, which can serve as the central step. In this latter role, it directly generates fermentable sugars, functional compounds, or biorefinery substrates. These routes and their products are summarised in Fig. 8.

Biological pretreatment partially degrades cellulose and modifies fibre structure. It generates low-molecular-weight soluble compounds that facilitate subsequent conversions.^153–155^ Thermophilic microbial consortia represent an environmentally favourable and energy-efficient alternative to conventional physicochemical methods. They operate between 50 and 60 °C without aggressive reagents.^156^

Zhang *et al.*^72^ demonstrated the effectiveness of this approach. They applied a thermophilic cellulolytic consortium to cassava residues combined with distillery wastewater. The wastewater presented high organic load (COD 24,580 ± 258 mg L^−1^), total nitrogen of 288.96 ± 12.34 mg L^−1^, total solids of 19.39 ± 0.03 g L^−1^, volatile solids of 14.12 ± 0.11 g L^−1^, and pH of 4.23 ± 0.02. After only 12 hours of pretreatment, methane production reached 259.46 mL g^−1^-VS. This represented a 96.63% increase relative to the control. The effect was attributed to increased FPase, CMCase, and xylanase activities. Solubilisation of approximately 60% of organic matter was also observed. The thermophilic consortium secretes endoglucanases, exoglucanases, β-glucosidases, xylanases, mannanases, and arabinofuranosidases. These enzymes convert structural polysaccharides into oligosaccharides and monosaccharides. Their release stabilises pH between 6.8 and 7.5, which is ideal for methanogenesis.

The advantages of biological pretreatment include low energy consumption and reduced pollutant generation. Prasad *et al.*^156^ consider it environmentally and economically favourable compared to conventional methods. The use of microbial consortia eliminates corrosive reagents. It also reduces the formation of thermal or chemical degradation inhibitors. Integration with anaerobic digesters is facilitated, as intermediate products are biochemically compatible.^135^

However, biological pretreatment has significant limitations. Conversion rates are relatively slow, often requiring over 12 hours of residence time. Chemical or thermochemical methods achieve comparable results in minutes. Process efficiency depends on strict control of temperature, pH, oxygen content, and microbial community composition.^157^ Contamination by undesirable microorganisms remains a concern in semi-continuous or long–duration processes. The lack of robust validation at industrial scale limits extrapolation from laboratory or pilot results. These challenges indicate that further advances are needed for reliable commercial biorefinery applications.^135,156^

Enzymatic pretreatment selectively degrades specific fractions without damaging components of interest. For starch-rich biomasses such as cassava bagasse, amylases remove the starchy fraction and expose the cellulosic structure. Panyasiri *et al.*^68^ demonstrated a substantial biomass transformation using this strategy. Starch content was reduced from 61.60% to 7.20%. α-cellulose content simultaneously increased from 19.27% to 50.45%. After bleaching and high-pressure homogenisation, cellulose nanofibrils of 15–30 nm were obtained. These presented higher crystallinity and thermal stability due to the selective removal of amorphous components.

This principle of selective cell wall degradation was also explored by Poonsrisawat *et al.*^158^ Different cassava substrates were tested, including fresh roots, chips, and pulp. A multienzyme cocktail produced by Aspergillus aculeatus was applied, containing endoglucanase, xylanase, polygalacturonase, β-glucanase, mannanase, and accessory activities. The synergistic action on pectins, hemicelluloses, and cellulose reduced viscosity by over 90% in fresh roots. Reductions of up to 92.9% were observed in fibrous pulp. No inhibitors such as furfural or HMF were generated. Ethanol concentrations near 19.65% v/v were reached *via* the thermal process, confirming operational and energetic gains from the enzymatic route.

The efficiency of enzymatic pretreatment on starchy cassava substrates was also shown in advanced biofuel production. Lépiz-Aguilar *et al.*^159^ used cassava flour hydrolysed by α-amylase and β-glucoamylase as substrate for ABE fermentation. *Clostridium beijerinckii* BA101 was used in a 5 L bioreactor. Results showed 25.71 g L^−1^ of butanol and 37.01 g L^−1^ of total ABE, with a productivity of 0.446 g L^−1^ h^−1^ at 83 hours. In a prior study, enzymatic saccharification outperformed HCl pretreatment (1 M, 121 °C). The more prolonged saccharification provided greater sugar release. Up to 31.59 g L^−1^ of butanol and 44.49 g L^−1^ of ABE were obtained in the best condition (40 °C, 60 g L^−1^), without generating inhibitory by-products.

When the substrate is predominantly lignocellulosic, isolated enzymatic pretreatment encounters structural limitations. Yu *et al.*^160^ compared 12 pretreatment methods applied to cassava starch factory residue, composed of 56.83% hemicellulose, 16.93% cellulose, and 4.41% lignin. Enzymatic treatment with hemicellulase and cellulase favoured oligosaccharide production (22 152 mg L^−1^). The highest total sugar yields (43 196 mg L^−1^; 51.84%) were achieved by combining 1% H_2_SO_4_ with hemicellulase and cellulase. This demonstrates synergy between chemical structural opening and enzymatic hydrolysis. In contrast, dilute acid alone (0.5% HNO_3_, 160 °C, 10 min) maximised monosaccharide formation (25 827 mg L^−1^), but at the cost of generating inhibitors. Integration of chemical and enzymatic pretreatments represents the most promising strategy. It maximises fermentable sugar release without the environmental and operational costs of aggressive chemical reagents alone.

### 5.5. Enzymatic hydrolysis

Beyond its role as a pretreatment, enzymatic hydrolysis can serve as the core of independent valorisation routes. These routes target fermentable sugars, functional compounds, or biorefinery substrates. One particularly relevant frontier is conducting hydrolysis below the gelatinisation temperature of starch. This eliminates the cooking step and substantially reduces process energy consumption. Uthumporn *et al.*^151,161^ showed that the combined application of α-amylase and glucoamylase at 35 °C for 24 hours achieved 35.4% dextrose equivalent conversion. Solubility increased from 3.2% to 5.3%. Peak viscosity decreased from 1846.8 to 1531.2 cP.

In a systematic comparison of roots and tubers, Rocha, Carneiro and Franco^161^ identified cassava starch as the most susceptible to bacterial α-amylase. After 48 hours at 37 °C, 20.9% hydrolysis was achieved. This was significantly higher than potato starch (5.9%). Amylose content decreased from 19.9% to 16.2%. Thermal properties remained practically unchanged. These results position cassava starches as attractive candidates for low-cost, low-impact hydrolysis routes.

Chen *et al.*^106^ reported severe surface erosion of cassava starch granules after enzymatic hydrolysis. Hydrolysis generated pores, cracks, and internal cavities connected to the granule interior. Combined α-amylase and glucoamylase treatment at 45 °C for 16 h increased specific surface area 10.7-fold, from 2.39 to 25.58 m^2^ g^−1^. Preferential hydrolysis of amorphous regions intensified XRD peaks, indicating increased relative crystallinity. Transition enthalpy increased from 11.8 to 16.4 J g^−1^, reflecting greater organisation of remaining crystalline domains.

Physical conditions prior to hydrolysis also determine enzymatic accessibility. Wu *et al.*^162^ showed that moderate heating of cassava starch to 70 °C, followed by rapid cooling to 50 °C before adding γ-CGTase, promoted controlled granule swelling. Viscosity decreased from 2456 to 23 cP. γ-cyclodextrin conversion reached 22.92% at starch concentrations up to 30%. Average molecular mass decreased from 3.815 × 10^8^ to 2.5 × 10^6^ g mol^−1^, and short chains (DP < 6) increased. This expanded the contact surface for enzymatic action without requiring complete gelatinisation, representing an energetically advantageous strategy.

The combination of physical pretreatment with enzymatic hydrolysis also opens relevant research frontiers. Arroyo-Dagobeth *et al.*^163^ investigated dual modification by Heat-Moisture Treatment (HMT) followed by enzymatic hydrolysis. Individual treatments with α-amylase, amyloglucosidase, and pullulanase were applied to binary mixtures of cassava and yam starch (70 : 30). Sequential treatment produced changes not achievable by either method alone. Pullulanase cleaves α-1,6 bonds of amylopectin and acts as a debranching enzyme. It raised apparent amylose content from 22.03% to 22.85% and water absorption capacity to 117.34%. It simultaneously reduced retrogradation tendency. This property is directly relevant to anaerobic digestion, where starch recrystallisation limits biodegradability.

Varongchayakul *et al.*^101^ combined Liquid Hot Water (LHW) pretreatment with enzymatic hydrolysis using Cellic® CTec2 cellulase at 37 °C for 72 hours. Glucose concentrations between 387 and 582 mg/gTS were obtained. Production rates ranged from 8.42 to 13.18 mg/gTS h, more than double those of untreated pulp (4.23–7.93 mg/gTS h). Total fermentable sugar conversion reached 87–99%, outperforming all compared approaches. These included acid routes and exclusively enzymatic strategies. However, a portion of more resistant cellulose remained unhydrolyzed. This indicates the need for complementary pretreatments or more specific enzymes for further process improvement.

Enzymatic hydrolysis can also target functional compounds of high added value, without primary focus on fermentable sugars. Pattarapanawan *et al.*^104^ investigated the sequential combination of pyrodextrinisation and enzymatic hydrolysis. Pyrodextrinisation is a low-moisture reaction at 140–200 °C with an acid catalyst. Subsequent hydrolysis with thermostable α-amylase and amyloglucosidase converted residual cassava pulp starch into resistant maltodextrin (RMD). The soluble fraction increased from 3.2 to 25.8 g/100 g. Crystallinity decreased from 24.5% to 15.3%. The product showed a prebiotic index of 0.64 for *Lactobacillus reuteri*, sustaining microbial growth for 72 hours, in contrast with glucose. This demonstrates that combining thermochemical modifications with enzymatic hydrolysis can produce functional profiles unachievable by any single method. It extends cassava pulp valorisation beyond biofuels toward circular bioeconomy models.

Gonzalez *et al.*^164^ demonstrated full integration of enzymatic hydrolysis with bioethanol production from cassava lignocellulosic and starchy fractions. Stem and peel were simultaneously evaluated as substrates in a unified process. For the stem, sequential pretreatment with 1.09% H_2_SO_4_ followed by 4% NaOH enriched the cellulosic fraction from 34.66% to 75.26%. Hemicellulose removal reached 61% and lignin removal reached 88%. Hydrolysis with Cellic CTec2 under optimised conditions (SL = 174.44 g L; EL = 25 mg g^−1^) yielded 116.89 g per L glucose with 80.19% hydrolysis efficiency.

For the peel, starch content reached 62.36%. Liquefaction with α-amylase at 90 °C followed by saccharification with glucoamylase at 55 °C produced 211.60 g per L glucose, with 61.10% yield. Integrated fermentation with *Saccharomyces cerevisiae* yielded 103.74 g per L ethanol, 92.2% efficiency, and 2.21 g L^−1^ h^−1^ productivity. These results confirm the technical viability of jointly valorising cassava residual fractions in second-generation biorefineries.^164^

Amylase pretreatment effectively solubilizes starch, exposes cellulosic microstructure, and enables higher value-added products. However, combining chemical and enzymatic pretreatments maximizes fermentable sugar release while reducing environmental and operational drawbacks of aggressive chemicals.

Despite its high selectivity and low inhibitor formation, enzymatic hydrolysis remains one of the major cost contributors in biomass conversion processes. Its performance is strongly influenced by enzyme loading, substrate accessibility, and product inhibition effects. In highly lignified cassava residues, complementary pretreatments are often required to disrupt the lignocellulosic matrix and improve enzyme accessibility. The economic feasibility of large-scale applications also depends on enzyme cost reduction and efficient recycling strategies.

**Fig. 9:**
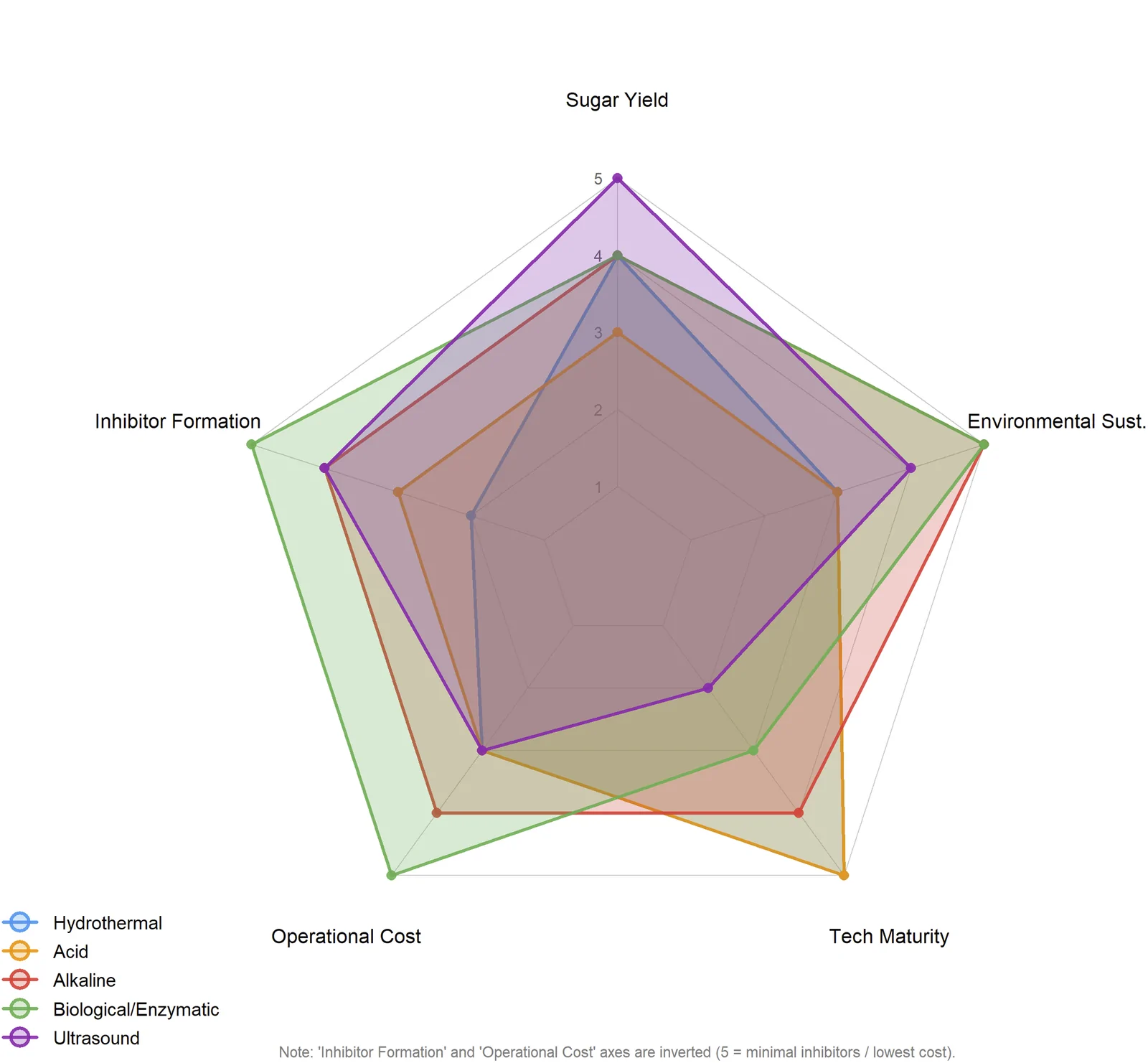
Comparative assessment of five pretreatment strategies for lignocellulosic cassava waste across sugar yield, inhibitor formation, cost, technological maturity, and environmental sustainability, based on literature-derived estimates.

Representative quantitative performance metrics for cassava residue pretreatment and valorization are summarized in Table 2, illustrating the variability in product yields, conversion efficiencies, and inhibitor formation across biomass types and operating conditions. These indicators complement the semi-quantitative comparison presented in Fig. 9, which assesses sugar yield, inhibitor formation, processing cost, technological maturity, and environmental sustainability across the main strategies reviewed herein. The scoring criteria and methodology are detailed in Supplementary Table S1.

**Table 2:** Quantitative performance metrics reported for representative cassava residue pretreatment and valorisation strategies

Reference	Biomass	Pretreatment/process	Optimized conditions	Main product	Performance metrics	Inhibitor formation
133	Cassava residues	Thermal-dilute sulfuric acid pretreatment (TDSA)	157.8 °C, 2.99% H_2_SO_4_, 20.15 min	Methane	248 mL CH_4_ g^−1^ VS (+56.96% compared with control)	Furanic compounds (HMF + furfural) up to 2.05 g L^−1^ under severe conditions
72	Cassava residues + distillery wastewater	Thermophilic microbial consortium	55 °C, 12 h	Methane	259.46 mL CH_4_ g^−1^ VS (+96.63% compared with control)	Acetic, propionic and butyric acids accumulated up to 10.6 g L^−1^
81	Cassava peels	Soaking assisted thermal pretreatment (SATP)	69.6 °C soaking, 2.57 h, 3.68% HCl, 5 min autoclaving, 9.65% solids	Reducing sugars	89.80 ± 2.87 g L^−1^; 0.93 ± 0.03 g g^−1^ peel; 90.79% recovery	Acetic acid: 260.52 ng g^−1^; levulinic acid: 147.97 ng g^−1^; formic acid: 85.89 ng g^−1^; HMF: 62.34 ng g^−1^; furfural: 53.70 ng g^−1^
105	Cassava stem	Sequential acid-alkaline pretreatment followed by enzymatic hydrolysis	174.4 g L^−1^ solids, 25 mg enzyme protein g^−1^ cellulose	Glucose	116.9 g L^−1^ glucose; 80.2% hydrolysis efficiency	Not reported
105	Cassava peel	Liquefaction and saccharification with α-amylase and glucoamylase	500 g L^−1^ solids; 40 µL g^−1^ α-amylase; 30.7 µL g^−1^ glucoamylase	Glucose/Ethanol	211.6 g L^−1^ glucose; 61.1% glucose yield; 103.7 g L^−1^ ethanol; 92.2% fermentation efficiency	Not reported
101	Cassava pulp	Liquid hot water (LHW) pretreatment followed by enzymatic hydrolysis	187 °C, 7 min, 5% solids	Glucose	266–295 mg glucose g^−1^ TS; up to 99% theoretical sugar conversion	Furfural: 32–39 mg L^−1^; HMF: 29–36 mg L^−1^

As shown in Fig. 9, hydrothermal and acid pretreatments share the highest technological maturity scores.^114,120^ Both have been validated at industrial scale. Acid pretreatment achieves the highest sugar yield among all strategies.^113,133^ However, it generates significant inhibitor formation under severe conditions, limiting downstream bioconversion. Alkaline and biological/enzymatic pretreatments achieve the highest environmental sustainability scores.^139,150^ Biological/enzymatic methods also present the lowest operational costs and minimal inhibitor formation. However, slow kinetics restrict their large-scale deployment.^156^ Ultrasound achieves the highest sugar yield score among all methods. Nevertheless, low technological maturity and moderate operational costs limit its current applicability.

## 6. Biotechnological methodologies

### 6.1. Fermentation

**Fig. 10:**
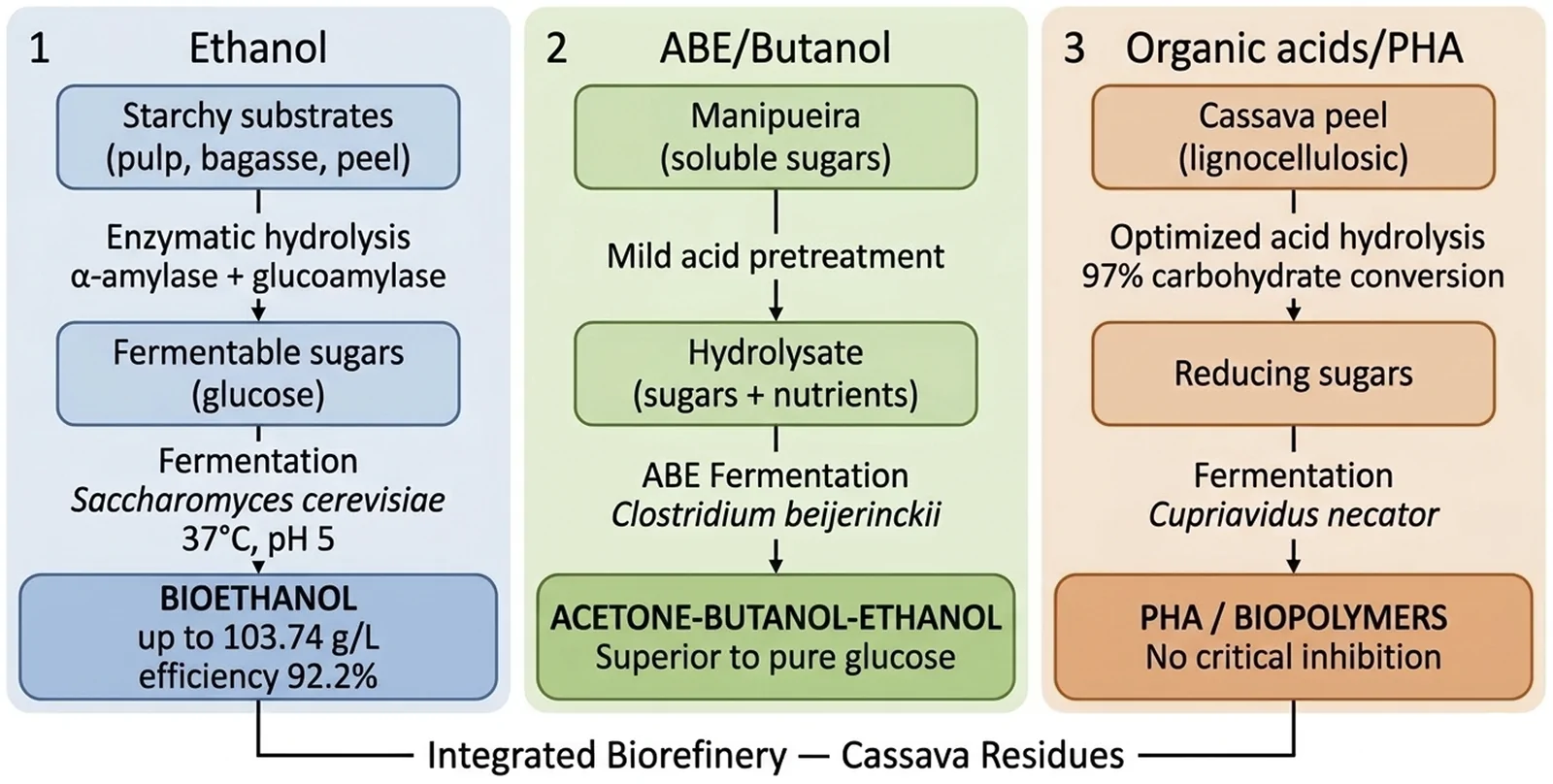
Main fermentation routes applied to cassava production chain residues, illustrating substrates, microorganisms and products obtained. Prepared by the author based on Chogi *et al.*,^109^ Hierro-Iglesias *et al.*^166^ and Gonzalez *et al.*^164^.

The fermentative conversion of cassava residues involves diverse substrates, microorganisms, and products, and depends directly on pretreatment and hydrolysis efficiency. Starchy and lignocellulosic residues show high fermentative reactivity when processed to release fermentable carbohydrates. Efficient hydrolysis and acidification of cassava bagasse and pulp by specialized microbial consortia have been reported.^165^ Similar performance occurs after appropriate saccharification of starch or fiber-rich substrates, yielding high glucose levels and subsequent ethanol or metabolite production. Optimized pretreatment and hydrolysis strategies, including microwave-assisted acid hydrolysis, enhance fermentative performance by maximizing sugar release and minimizing inhibitors.^20^ The main fermentation pathways for cassava residues are summarized in Fig. 10 and detailed in the following sections.

In general, the literature demonstrates that cassava residues exhibit good fermentative performance when carbohydrates are bioavailable and the formation of inhibitory compounds is controlled. However, it is observed that many studies still focus on pretreatment steps, while a more limited number evaluate complete chains including fermentation. When fermentation is conducted, the results confirm the high versatility of cassava as a biological substrate.^165,167^

Among solid residues, cassava peel, pulp, and bagasse hydrolysates have been successfully used in fermentation processes. Cassava peel hydrolysates enabled polyhydroxyalkanoate production by *Cupriavidus necator* without inhibition, as furfural and HMF remained below critical levels.^166^ Other studies report efficient sugar release from these residues, though fermentation was not always evaluated. High-energy milling generated starchy hydrolysates readily assimilated by *Escherichia coli* without inhibitor formation.^168^ Combined hydrothermal pretreatment and enzymatic hydrolysis of starch residues produced up to 706 g kg^−1^ glucose.^169^ Microwave-assisted hydrolysis enhanced sugar release and reduced inhibitors, improving fermentative performance.^20^ Ultra-high-pressure water-jet pretreatment increased cellulose saccharification up to fivefold without inhibitors, although fermentation was not assessed.^170^ Overall, these findings confirm the high fermentative potential of cassava residues, even when studies focus primarily on pretreatment and hydrolysis stages.

The integration of cassava residues as co-substrates has been explored in mixed lignocellulosic fermentation systems. In glycerol-based organosolv processing of corn cobs, cassava supplied fermentable carbohydrates, reduced phenolic and furan toxicity, and enhanced butanol production by *Clostridium acetobutylicum*.^171^ This behavior highlights cassava residues as stabilizing substrates in integrated fermentation processes.

Among liquid wastes, cassava wastewater is the most extensively studied from a fermentative perspective. This effluent contains high concentrations of soluble sugars and generates negligible inhibitor levels under mild acid pretreatment. Under these conditions, cassava wastewater enabled ethanol and butanol production exceeding that obtained from pure glucose in ABE fermentations by *Clostridium beijerinckii*.^109^ These results demonstrate the high suitability of cassava processing liquid streams for industrial fermentative applications.

In fermentative processes targeting nutritional improvement and detoxification, *Citrobacter freundii* and *Proteus vulgaris* showed high cellulolytic and cyanolytic activity on cassava peel–leaf mixtures. Fermentation reduced crude fiber from 21.96% to 9.24% and HCN from 155.82 to 31 ppm, while increasing crude protein to 17.67%.^172^ These effects result from synergistic cellulase and β-glucosidase action degrading structural polysaccharides and cyanogenic glycosides. Additionally, *Bacillus* isolates from cassava waste landfill soil exhibited cellulase activities of 2.32–7.58 U/mL and cellulolytic indices of 0.81–2.06, highlighting their potential as microbial inoculants for feed-oriented fermentative processes.^172,173^

The fermentative valorisation of cassava residues into high-value bioproducts has gained increasing attention. Acid-hydrolysed cassava pulp subjected to dark fermentation produced 12.39 ± 0.8 L H_2_ kg^−1^ waste with 99.89% purity under optimized pH and HRT conditions.^174^ High hydrogen yields were partly attributed to CO and CO_2_ dissolution, shifting equilibrium toward H_2_. Cassava processing waste also served as a carbon source for PHBV biosynthesis by *Paracoccus* sp. KKU01, reaching 33.9 g L^−1^ with controlled 3-hydroxyvalerate content.^175^ In another route, *Aureobasidium pullulans* produced pullulan *via* solid-state fermentation on cassava residue, achieving 6.45 g L^−1^ after factorial optimization.^176^ Collectively, these studies demonstrate that cassava residues can be converted into biopolymers and energy vectors suitable for biomaterials, packaging, pharmaceutical, and clean energy applications.

Cassava residues show high fermentative potential when pretreatment, hydrolysis, and fermentation are effectively integrated. Starchy residues respond rapidly and support articulated experimental chains already approaching process integration. Lignocellulosic residues require stricter pretreatment control, and systematic integration studies remain scarce for recalcitrant fractions like peels and leaves.

### 6.2. Anaerobic digestion

Anaerobic digestion is a key biotechnological strategy for energy recovery from cassava residues, converting organic matter into biogas while reducing pollutant load and enhancing circularity.^9,11^ Process efficiency depends on substrate composition and operating conditions, as inhibitory compounds may disrupt acidogenic and methanogenic stages, requiring careful optimization.^177^ Anaerobic digestion relies on synergistic interactions among hydrolytic, acidogenic, acetogenic bacteria, and methanogenic archaea. Hydrolysis is often rate-limiting for lignocellulosic substrates, while methanogenesis is highly sensitive to VFAs, sulfides, ammonia, and pH variations.^178,179^ Cassava effluents, pulp, and lignocellulosic by-products exhibit high biochemical methane potential but require targeted strategies for efficient digestion. These include pretreatments, co-additives, and multi-phase digestion systems to overcome hydrolytic limitations, reduce inhibitors, and improve process stability.^107,180^ The following sections address these strategies, relevant substrates, and the main inhibitors reported in the literature.

Among studied substrates, cassava wastewater stands out for high organic load and biodegradability, with BOD_5_/COD ratios above 0.45, indicating suitability for anaerobic digestion^.^ Nonetheless, this effluent often exhibits elevated cyanide concentrations and high VA/TA ratios, posing risks of acidification and microbial inhibition.^181^ Cyanide removal by solar evaporation followed by pH adjustment enabled efficient digestion without complex pretreatments. Co-digestion of 80% cassava wastewater with 20% primary sewage sludge achieved 81.41% methane within 48 days under ambient conditions.^182^ Co-digestion with other wastes also stabilized pH and diluted toxic compounds. In two-phase biodigestion, glycerol supplementation produced 30.8 mL H_2_ g^−1^ RCOD and 104.5 mL CH_4_ g^−1^ RCOD, enhancing energy efficiency.^107^

The hydrolysis stage remains the main bottleneck for lignocellulosic cassava residues during anaerobic digestion. Biological pretreatment using thermophilic microbial consortia increased methane yield by 96.6%, attributed to rapid organic matter solubilisation during early incubation.^72^ Similarly, the EHA4 consortium degraded up to 100% of cassava pulp within four days, producing butyrate- and acetate-rich VFAs suitable for methanogenesis.^165^ These studies demonstrate that specialized consortia can reduce reliance on chemical pretreatments and overcome hydrolytic limitations. However, strict control of exposure time and pH is critical, as prolonged pretreatment reduces soluble carbon and acidic conditions intensify VFA inhibition.^72,165^ Complementarily, thermomechanical extrusion disrupted starch semicrystallinity, increasing methane production by 42% and shortening the adaptation phase from 41 to 23 days.^108^

pH control is a critical factor in all anaerobic digestion systems. A pH drop indicates volatile fatty acid accumulation, including acetate, propionate, butyrate, and lactate, the main process inhibitors. Cassava starch polymer digestion above 10 g L^−1^ caused VFA accumulation over 3000 mg L^−1^, dominated by propionate and lactate, leading to severe methanogenesis inhibition.^183^ In a three-stage UASB system treating starch effluent, organic loadings above 15 kg COD·m^−3^ d^−1^ caused acidification, with pH falling to 4.95–6.05. This triggered methanogenic collapse. However, within appropriate loading limits, the system achieved COD removals exceeding 92% and a methane yield of 328 mL CH_4_ g^−1^ COD applied.^184^ Recycling methanogenic effluent into initial reactors buffered pH and reduced external alkalizing demand. However, it also compromised hydrogen production by introducing methanogens into the acidogenic phase.^184^ Adding sodium bicarbonate provided strong buffering capacity and significantly enhanced methane production. Mesophilic temperature shifts exerted comparatively minor effects on overall process performance.^107^

Physical separation between acidogenic and methanogenic stages through two-phase digestion is the main strategy to overcome inhibition problems. This approach creates specific environments for each microbial group. The acidogenic phase operates at pH 5.0–6.0 and 50–60 °C. The methanogenic phase requires pH 6.8–7.4 and 35–38 °C. This separation allows rigorous control of acid formation and avoids competition between microbial groups. Energy gains exceeding 40% were reported compared to single-phase systems.^185^ When applied to cassava starch combined with sugarcane bagasse, the two-stage arrangement reduced the total digestion time by more than 78%. This increased the overall methane production rate by 305%, and produced biogas with approximately 82% CH_4_, while generating hydrogen as a co-product in the acidogenic phase.^108^ At the biorefinery scale, conventional anaerobic digestion (CAD) applied to cassava wastewater produced biogas with up to 75% methane. In comparison, modified acidogenic digestion (MAD) produced over 100 mg mL^−1^ VFAs, and integrating these routes cut payback by 18%, raised net present value 75%, and reduced impacts 54.4%.^111^

Even after anaerobic digestion, recalcitrant compounds such as melanoidins and lignin-derived phenols persist, compromising final effluent quality. These high-molecular-weight fractions tend to accumulate, requiring complementary physicochemical polishing steps. Coagulation with FeCl_3_ removes up to 95% of color and 78% of dissolved organic carbon *via* complexation and charge neutralisation.^186^ Integration of anaerobic digestion with microalgal systems has been proposed for effluent polishing and nutrient recovery. In such systems, biogas CO_2_ supports microalgal growth, while digestate supplies nitrogen and phosphorus, enabling circular valorisation of cassava waste.^110^ Additionally, deficiencies of micronutrients such as Fe, Zn, and Mo can limit methanogenic activity in starch effluent digestion systems.^184^

The efficiency of anaerobic digestion depends on biotechnological strategies that modulate hydrolysis, control metabolic pathways, and mitigate inhibitors such as VFAs, cyanide, and rapid acidification.^11,181^ Integrating biological, thermomechanical, and physicochemical pretreatments with optimized reactor configurations and biorefinery concepts enables more efficient and stable processing systems. These integrated approaches support the production of biogas, biohydrogen, VFAs, and other high-value bioproducts.^111,185^ In this context, anaerobic digestion emerges as a strategic pathway for biomass-based energy transitions, particularly in regions where cassava holds strong socioeconomic relevance.

**Fig. 11:**
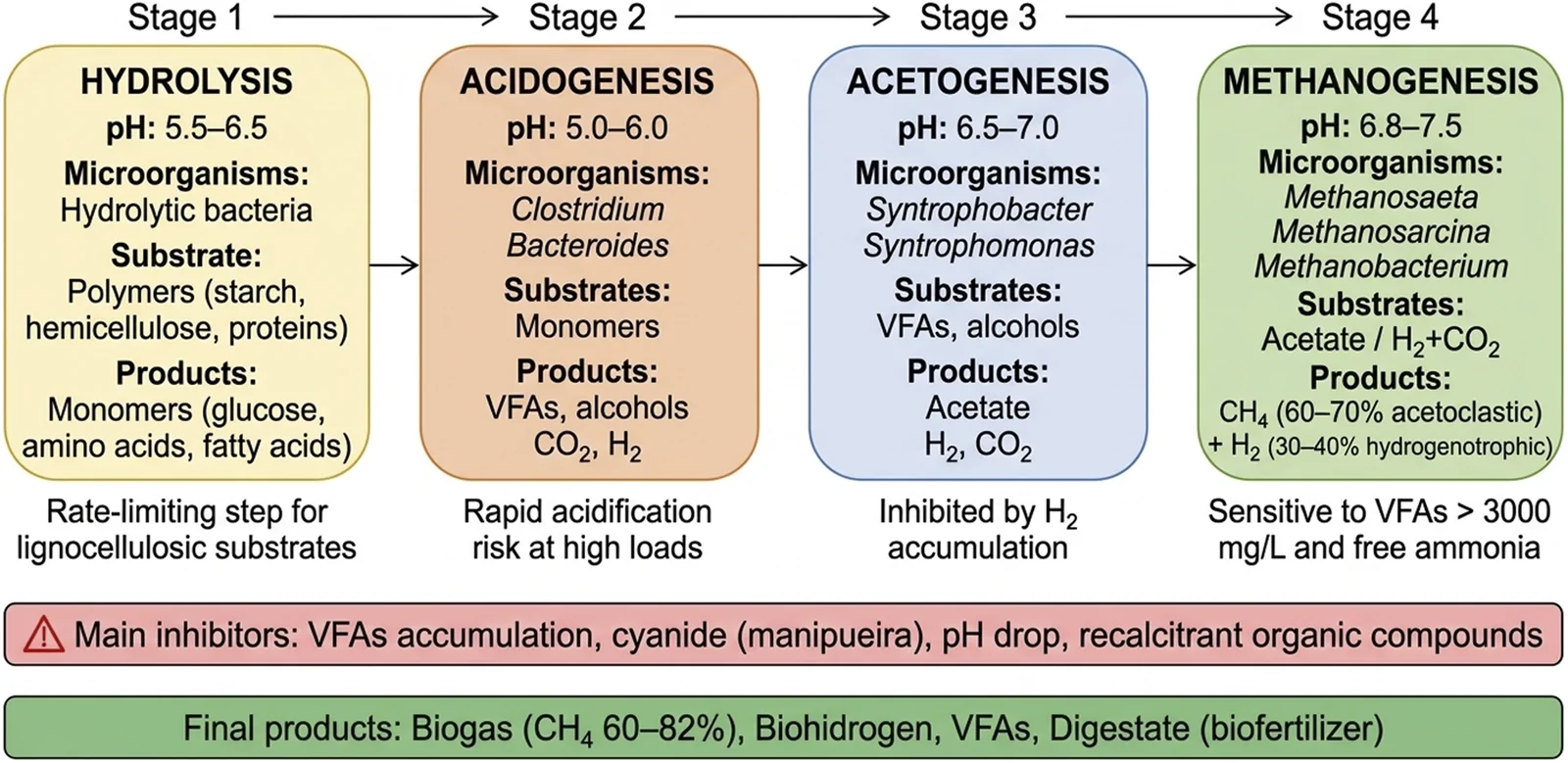
Interdependent biochemical steps of anaerobic digestion of cassava residues, indicating pH ranges, responsible microorganisms, intermediate products, and main operational inhibitors. Prepared by the author based on Chen *et al.*,^178^ Mata-Alvarez *et al.*^179^ and Costa *et al.*.^181^

Fig. 11 illustrates these four biochemical steps in an integrated way, highlighting the pH ranges, the main microorganisms involved, and the critical inhibitors identified for cassava residues.

## 7. Emerging technologies

Emerging technologies such as ultrasound, non-thermal plasma, and integrated bioreactors provide intensified mechanisms that overcome key limitations of conventional biomass processing methods.^187^ These approaches often operate under milder conditions, generate fewer inhibitory compounds, and offer higher selectivity for target fractions in complex systems. Despite increasing interest, studies applying ultrasound or plasma to cassava residues remain scarce. A Scopus search using “ultrasonic pretreatment” OR “plasma pretreatment” AND “cassava” identified only six studies directly addressing cassava-derived feedstocks. These studies cover pretreatment, detoxification, functional modification, and biofuel production contexts. This limited number highlights a significant research gap and underscores the need to explore these technologies for cassava waste valorisation. Table 3 summarizes the identified studies, illustrating their temporal distribution and diversity of applications.

**Table 3:** Studies involving ultrasound, plasma, and cassava identified in the literature

Authors	Title	Year	Journal title
Preethi *et al.*^188^	Ultrasonic-alkaline-thermal pretreatment on cassava sago waste for energy efficient biomethane production	2026	*Process Biochemistry*
Hu *et al.*^189^	Effect of starch content and ultrasonic pretreatment on gelling properties of myofibrillar protein from Lateolabrax japonicus	2023	*Food Frontiers*
Sabiani *et al.*^190^	Kinetic evaluation of sonicated food waste in continuously stirred tank reactor	2022	*Water Science and Technology*
Zhong *et al.*^191^	Effect of ultrasonic pretreatment on eliminating cyanogenic glycosides and hydrogen cyanide in cassava	2021	*Ultrasonics Sonochemistry*
Shanthi *et al.*^192^	Effect of surfactant assisted sonic pretreatment on liquefaction of fruits and vegetable residue: Characterisation, acidogenesis, biomethane yield and energy ratio	2018	*Bioresource Technology*
Nitayavardhana *et al.*^102^	Ultrasound pretreatment of cassava chip slurry to enhance sugar release for subsequent ethanol production	2008	*Biotechnology and Bioengineering*

Analysis of Table 3 reveals that ultrasound is the most explored technology in association with cassava. Its applications range from the intensification of saccharification^102^ to the detoxification of cyanogenic glycosides^191^ and the functional modification of starches in protein systems.^189^ Furthermore, none of the studies involving plasma were identified with the string, reinforcing the emerging of this technology in the context of cassava.

**Fig. 12:**
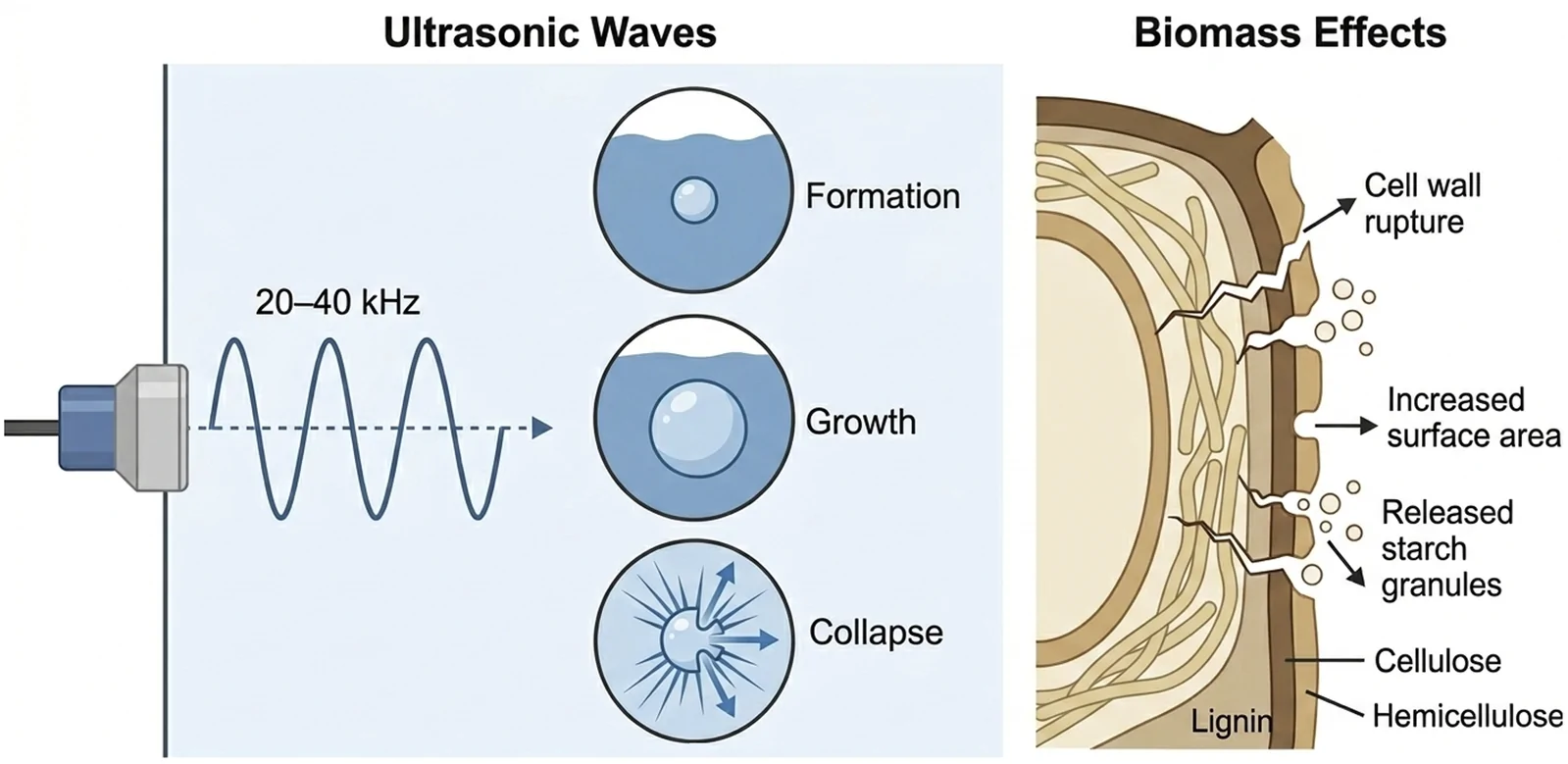
Schematic representation of the acoustic cavitation mechanism induced by low-frequency ultrasound (20–40 kHz) and its effects on lignocellulosic biomass. The left panel illustrates the propagation of ultrasonic waves through the transducer; the central panel details the nucleation, growth, and violent collapse phases of microbubbles; and the right panel demonstrates the resulting physical effects on the cell wall of the biomass, including microfibril rupture, increased porosity, and release of starch granules. Prepared by the author based on Preethi *et al.*,^188^ Hu *et al.*,^189^ Sabiani *et al.*,^190^ Zhong *et al.*,^191^ Shanti *et al.*,^192^ Nitayavardhana *et al.*.^102^

Among the technologies discussed, ultrasound has the largest volume of evidence applied to diverse biomasses, including cassava residues. Its central mechanism is acoustic cavitation, which involves the formation and violent collapse of microbubbles in a liquid medium (Fig. 12).

Ultrasound generates microjets, shock waves, and hydrodynamic shear that fragment particles, disrupt cell walls, reduce polymerisation degree, and increase enzymatic accessibility.^193,194^ These effects are intensified when ultrasound is combined with chemical agents, producing synergistic performance beyond isolated treatments. Xu *et al.*^193^ showed that ultrasound with dilute aqueous ammonia (1–4 wt%) increased enzymatic hydrolysis yields of corn cob (80.6%), corn stover (66.2%), and sorghum (56.9%). Partial delignification and morphological changes were confirmed by XRD and SEM analyses. Similarly, Ong *et al.*^195^ integrated ultrasound with NaOH and a deep eutectic solvent (ChCl:urea), achieving 47% lignin removal and 90.02% glucose yield from palm fronds. This approach increased surface area from 0.40 to 0.85 m^2^ g^−1^ and enhanced cellulose microfibril exposure.

As illustrated in Fig. 12, ultrasound effects on biomass are predominantly mechanical, resulting from asymmetric cavitation bubble collapse near surfaces. This collapse generates microjets exceeding 100 m s^−1^, fragmenting particles, rupturing cell walls, and disrupting semicrystalline starch structures. Nitayavardhana *et al.*^102^ confirmed this mechanism during ultrasonic pretreatment of cassava chip pulp. They observed up to 40-fold particle size reduction, extensive starch granule release, and a 180% increase in reducing sugars compared to untreated material. The authors demonstrated that cavitation-induced physical disintegration, rather than heating, is the dominant mechanism. Consequently, higher solids content (25% TS) improved energy efficiency, achieving ratios above three, supporting industrial integration in cassava ethanol plants. Despite starch gelatinising at 60–70 °C, ultrasound up to 90 °C does not markedly gelatinise or disrupt granules. Instead, ultrasound promotes protein unfolding and intensified protein–starch interactions, forming denser and more cohesive three-dimensional networks.^189^

In food safety and waste processing, ultrasound under mild conditions activates endogenous β-glucosidase in cassava, enhancing cyanide detoxification. At 45 °C, 81 W, and 10 min, β-glucosidase activity increased by 18%, removing 40.36% of free cyanide and 24.95% of cyanogenic glycosides. Ultrasound complemented conventional cooking, outperforming prolonged immersion when applied as a pretreatment.^191^ Further along the value chain, Preethi *et al.*^188^ evaluated industrial sago waste using a combined ultrasound–alkaline–thermal process (USAT). This sequential pretreatment increased organic matter solubilisation from <2% to 29.6% and yielded 303.4 mL CH_4_ g^−1^ VS consumed. USAT was the only strategy achieving positive net energy, with an energy ratio of 1.54. These findings demonstrate that integrated ultrasound-based pretreatments are critical for starch-rich substrates. Overall, ultrasound can be strategically applied across cassava processing, from detoxification to fermentable sugars, biomethane, and functional ingredients.

The efficiency of ultrasound as pretreatment has also been evaluated in studies with other biomasses and food waste, with potential for generalisation to cassava. Sabiani *et al.*^190^ applied ultrasound at 20 kHz for 10 minutes to mixed food waste before anaerobic digestion in a CSTR reactor. They observed increases of 18.99% in methane production rate and 23.86% in overall yield, with excellent fit to the Monod kinetic model (*R*^2^ = 0.9958). Hence, sonication simultaneously favored microbial growth and substrate utilisation under mesophilic conditions. Rouabhia *et al.*^196^ expanded this model by proposing an integrated biorefinery strategy combining ultrasound, dark fermentation, and enzymatic hydrolysis for four distinct biomasses. The study shows that separating liquid and solid fractions after sonication enables multiple simultaneous valorisation pathways. They achieved H_2_ yields up to 20.8 mL per g dry matter, VFA concentrations up to 5.37 g H-Ac per L, and sugar conversion up to 47%. Orange peel, with low lignin and high soluble fibre, provides a useful analogue for certain cassava residue fractions. This integrated bioreactor model represents an important conceptual evolution, as it maximizes energy and biochemical return per unit of biomass processed. It can also reduce effluent generation while increasing the circularity of the process.

Jo *et al.*^197^ investigated sequential ultrasound–hydrothermal pretreatment using pine and yellow poplar as feedstocks. Initial ultrasound with H_2_O_2_ induced structural cracking and partial lignin removal, enhancing subsequent hydrothermal effectiveness. The hydrothermal step at 170–190 °C intensified hemicellulose autohydrolysis, achieving cellulose-to-glucose conversion up to 89.48%. Electron microscopy, immunolocalisation, and lignin autofluorescence confirmed progressive xylan and mannan removal and increased cell wall porosity. These mechanisms support hybrid pretreatments for recalcitrant biomasses such as cassava peels and bagasse. Among oxidative routes, potassium ferrate (Fe(vi)) combined with ultrasound achieved up to 38.9% delignification and methane production of 203 mL per g VS.^198^ The sequential ultrasound–ferrate strategy further improved performance, reaching 37.9% lignin removal, SCOD/TCOD of 0.311, and methane yield of 234.9 mL per g VS.^199^ These results demonstrate that pretreatment sequence critically determines efficiency, a principle directly applicable to highly lignified cassava residues.

Recent advances in plasma technologies reveal strong complementarity with ultrasound, particularly for thermochemical pathways and structural modification of biopolymers.^200^ Dielectric barrier plasma generates high-energy electrons and reactive species, including hydroxyl radicals and ozone, during electrical discharge.^201,202^ These reactive species interact efficiently with lignocellulosic materials, promoting surface oxidation and chemical activation without corrosive reagents or high temperatures. Consequently, plasma treatments enable polymer modification while preserving bulk structure and minimizing deep degradation.^203^ Studies further demonstrate that dielectric barrier plasma induces partial lignocellulosic degradation, causing microstructural disorganisation and exposure of crystalline domains.^201,204^ These combined effects facilitate subsequent hydrolysis and conversion steps, supporting plasma integration into advanced pretreatment strategies.^204^

Hu *et al.*^103^ showed that cold plasma treatment of cellulose and starch significantly reduced crystallinity, polymerisation degree, and thermal stability while increasing anhydroglycoside yields during pyrolysis. Cellulose crystallinity decreased from 74.59% to 67.79%, accompanied by polymerisation reduction from 240 to 167 and increased carbonyl and carboxyl groups. These changes raised anhydroglycoside yields from 24.58% to 39.48%, evidencing enhanced thermochemical reactivity. The positive plasma response of starch is particularly relevant for cassava, given its high starch content in dry residues. This supports plasma application for improving thermochemical conversion of starchy cassava wastes.^102^ Karthikeyan *et al.*^205^ integrated microbubble plasma with the Fenton process, achieving 32% lignin removal from corn biomass. This integration increased biomethane production by 26% in batch assays and 16% in continuous systems under ambient conditions. Minimal inhibitor formation reinforced plasma technologies as promising complements in integrated valorisation routes for recalcitrant lignocellulosic biomasses.

Integrated bioreactors, in turn, represent a growing trend in process intensification, allowing traditionally separate steps, such as pretreatment, saccharification, and fermentation. These steps can occur in a coupled or sequential manner in the same reaction medium. One-pot systems based on ionic liquids^206^ and ultrasound-assisted alkaline deep eutectic solvents^195^ have been used to demonstrate this integration. Overall, it reduces operational costs, increases saccharification efficiency, and favors subsequent conversion into biofuels.^195,206^ In anaerobic digestion, CSTR reactors fed with sonicated substrates exhibit more favorable kinetics and greater operational stability, reinforcing the potential of integrating physical pretreatments and biological processes.^190^

Despite their promising performance, the industrial application of ultrasound and plasma technologies remains limited. Current evidence is largely restricted to laboratory-scale studies, particularly for cassava residues. Process optimization, reactor design, and long-term operational performance still require further investigation. In addition, the relatively low technological maturity of these approaches has limited their adoption in industrial biorefineries. Further pilot-scale studies are necessary to validate their practical applicability.

Taken together, these studies indicate that ultrasound, plasma, and integrated bioreactors form a coherent technological axis for biorefinery process intensification. For cassava residues with variable starch, fiber, lignin, and cyanogenic contents, these technologies overcome key limitations of conventional processing routes. Their application supports detoxification, enhanced hydrolysis, increased bioenergy yields, and high-value bioproducts, consolidating cassava as a strategic biomass for energy transition.

## 8. Gaps and future perspectives

Despite advances in understanding cassava waste valorisation, this review identifies significant scientific gaps requiring further investigation. Scopus searches revealed only six studies addressing ultrasound or plasma applications to cassava residues, far fewer than for other lignocellulosic biomasses.^102,188,207^ This limited number indicates that the potential of these emerging technologies for cassava remains largely unexplored. Technologies such as microwave-assisted hydrolysis, hydrothermal processes, supercritical fluids, deep eutectic solvents, ionic liquids, and plasma–ozone systems have shown substantial benefits for other biomasses. These benefits include improved digestibility, reduced inhibitor formation, and enhanced conversion to bioproducts such as single-cell protein, suggesting promising routes still unvalidated for cassava residues.^208^

Studies applying DBD and cold plasma to cassava bagasse, peels, and wastewater remain scarce, despite successful results achieved for cellulose, starch, and other lignocellulosic biomasses. Although Fenton–plasma integration is promising, plasma treatments involve high specific electrical energy demand, restricting economic feasibility to selected process configurations.^205,209^ This energy intensity represents a major barrier to industrial scale-up of plasma-based technologies.^103,196,198^ Another critical limitation is the lack of pilot- or industrial-scale validation for most advanced pretreatments. Approaches such as SATP, microwave treatment, and thermophilic microbial consortia often show strong laboratory performance but limited scalability ^81 156^.

The development of techno-economic studies and life cycle analyses specific to biorefineries based on these cassava residues is equally necessary. The commercial viability of integrated routes depends not only on technical performance but also on cost analysis, energy consumption, and environmental impact throughout the entire production chain.^111^

Comparative techno-economic analyses show that cassava requires lower minimum ethanol production scales than sugarcane, rice, or palm residues to ensure economic viability^210,211^. This characteristic reinforces cassava attractiveness for second-generation biofuels in small-to medium-scale applications. Cassava wastewater remains underexplored in integrated systems targeting simultaneous hydrogen, methane, and volatile fatty acid production, particularly in cassava-dependent regions.^14,107,181^ Developing cassava varieties with improved compositional traits can further enhance industrial performance. When coupled with optimized post-harvest strategies reducing physiological deterioration, residue quality is better preserved. Together, these advances position cassava as a strategic biomass for circular and low-carbon bioeconomies.

## 9. Conclusion

This review demonstrates that the cassava production chain generates heterogeneous residues, including peels, bagasse, leaves, stems, and wastewater, with underexploited biotechnological potential. This underutilisation is particularly evident in major African cassava-producing countries, which contribute less than 5% of global starch output. Bromatological characterisation highlights leaves with high crude protein (28.02%) and bagasse rich in residual starch (up to 64%) as promising biorefinery feedstocks. Among pretreatments, hydrothermal processing offers the best balance between efficiency, inhibitor formation, and technological maturity. Alkaline pretreatment excels in delignification, while biological and enzymatic routes are the most sustainable, despite kinetic limitations. Anaerobic digestion achieves methane yield increases exceeding 96% when coupled with appropriate pretreatments. Fermentation and enzymatic hydrolysis remain key pathways for producing ethanol, butanol, and organic acids. Emerging technologies, including ultrasound and cold plasma, show strong potential for process intensification and detoxification, though cassava-specific studies remain limited. Overall, integrating these routes within circular biorefineries represents a robust strategy to convert cassava waste into bioenergy and high-value bioproducts. Such approaches strengthen the sustainability of the cassava value chain and contribute to food and energy security in tropical regions.

## Author contributions

Tammires Lorena C. Santana: writing – review & editing, writing – original draft, funding acquisition. Jude A. Onwudili: writing – review & editing, conceptualisation, supervision. Carine Tondo Alves: writing – review & editing, conceptualisation, supervision. Taíse Bomfim de Jesus: writing – review & editing, supervision.

## Conflicts of interest

The authors declare that there are no conflicts of interest regarding the publication of this manuscript.

## Supplementary Material

RA-016-D6RA04237F-s001

## Data Availability

No experimental data were generated during this study. Fig. 1 and 4 were prepared using data obtained from publicly available databases (FAO and Scopus, respectively). The remaining figures were developed by the authors based on information compiled from previously published literature. All sources used in the preparation of the manuscript are cited in the reference list. Supplementary information: Table S1 provides the semi-quantitative scores used to compare pretreatment strategies for cassava lignocellulosic residues across five performance criteria, based on data compiled from the literature reviewed in this study. Higher scores for inhibitor formation and processing cost indicate lower inhibitor generation and lower costs, respectively. See DOI: https://doi.org/10.1039/d6ra04237f.
