# Supplementary material for: A comprehensive review of methods for valorisation and production of bioproducts from various cassava wastes
Source: RSC Adv. 2026 Aug 24;16(41):47421–52. doi: 10.1039/d6ra04237f (PMC13501378; doi:10.1039/d6ra04237f)
Supplement: Supplementary file 1 [file RA-016-D6RA04237F-s001.pdf]

Supplementary Information (SI) for RSC Advances.
This journal is © The Royal Society of Chemistry 2026

Supplementary Material

Supplementary Table S1. Semi-quantitative scoring of pretreatment strategies for cassava lignocellulosic residues across five performance criteria (scale: 1 = least favorable; 5 = most favorable). Scores were assigned based on qualitative and semi-quantitative data extracted from peer-reviewed studies reviewed herein. For Inhibitor Formation and Processing Cost, higher scores indicate lower inhibitor generation and lower cost, respectively, consistent with the inverted axes in Figure 9

Criterion	Hydrothermal	Acid	Alkaline	Biological/Enzymatic	Ultrasound
Sugar yield	4	3	4	4	5
Inhibitor formation	2	3	4	5	4
Processing cost	3	3	4	5	3
Technological maturity	5	5	4	3	2
Environmental sustainability	3	3	5	5	4
